# Single-Cell Chemical Proteomics (SCCP) Interrogates the Timing and Heterogeneity of Cancer Cell Commitment to Death

**DOI:** 10.1021/acs.analchem.2c00413

**Published:** 2022-06-22

**Authors:** Ákos Végvári, Jimmy E. Rodriguez, Roman A. Zubarev

**Affiliations:** Division of Physiological Chemistry I, Department of Medical Biochemistry & Biophysics, Karolinska Institutet, Biomedicum A9, Solnavägen 9, SE-171 77 Stockholm, Sweden

## Abstract

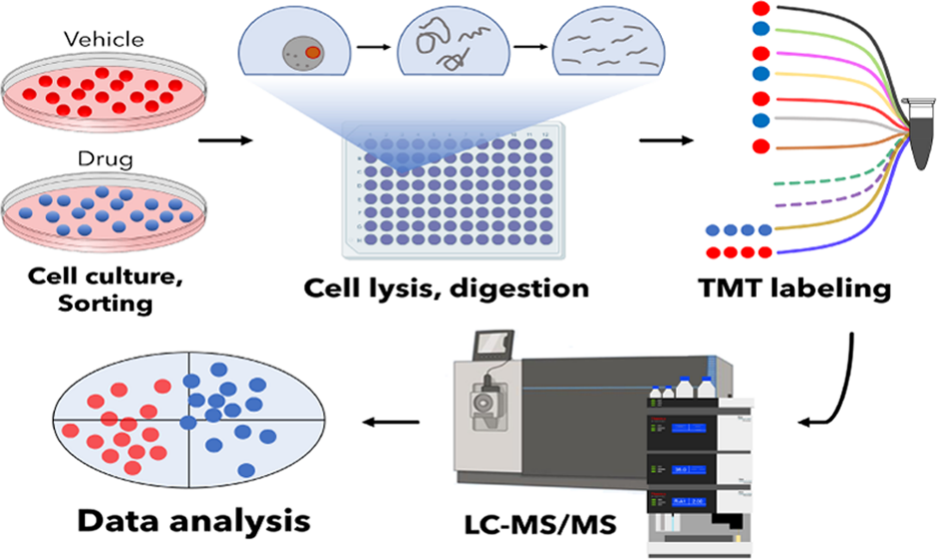

Chemical proteomics studies the effects of drugs upon a cellular proteome. Due to the complexity and diversity of tumors, the response of cancer cells to drugs is also heterogeneous, and thus, proteome analysis at the single-cell level is needed. Here, we demonstrate that single-cell proteomics techniques have become quantitative enough to tackle the drug effects on target proteins, enabling single-cell chemical proteomics (SCCP). Using SCCP, we studied here the time-resolved response of individual adenocarcinoma A549 cells to anticancer drugs methotrexate, camptothecin, and tomudex, revealing the early emergence of cellular subpopulations committed and uncommitted to death. As a novel and useful approach to exploring the heterogeneous response to drugs of cancer cells, SCCP may prove to be a breakthrough application for single-cell proteomics.

## Introduction

Chemical proteomics studies the effects of drugs on cellular proteomes with the purpose of deciphering the targets and mechanisms of action of these molecules.^1−3^ When sensitive cells are treated with toxic compounds for an extended period of time, mechanistic target proteins become significantly regulated, and their profiling provides the first hint on the compound’s targets and mechanisms of action.^4,5^ This approach has been employed in functional identification of targets by expression proteomics (FITExP),^6^ which laid the ground for the online chemical proteomics ProTargetMiner tool.^2^ In FITExP, cells are treated at an LC_50_ concentration for 48 h, by which time half of the cells die. The dying cells detach from the substrate (for adherent cell types) and are found floating on the flask surface. In the remaining surviving cells, the drug target’s expression level is significantly and specifically regulated up or down, which serves as a basis for drug target identification in FITExP. As an example, when cancer cells undergo treatment with methotrexate (MTX), the target protein dihydrofolate reductase (DHFR) becomes highly upregulated before the cells undergo programmed cell death.^7−9^ Interestingly, while the proteomes of the dying and surviving cells are very different (lending support to the notion that cell death is the ultimate case of cell differentiation), the drug target behaves in a similar manner in both types of cells.^2^

The adherent cells usually start losing their attachment to the surface after 24 h of treatment at LC_50_ concentration, but the decision to survive or dye must be made by the cell well before that.^1,10,11^ The intricate details of this decision-making process are of great scientific interest, as they possibly hold keys to the drug resistance mechanisms.^1,6^ These decision-making processes can only be studied at the single-cell level,^12^ while all so far reported chemical proteomics studies relied on bulk cell analysis.^13^ Cellular heterogeneity is currently analyzed routinely by single-cell transcriptomics,^14^ with mRNA levels assumed to be proportional to the protein expression levels. However, at any given moment, the concentration of both mRNA and proteins reflects the balance between their corresponding expression and degradation, and while mRNA transcription and protein expression are linked together rather well, the degradation processes for mRNA and proteins are completely decoupled. As a result, in the biological processes driven mostly by protein expression, mRNA levels provide an excellent proxy for protein concentrations, but this correlation seems to break down already at steady states of the cell.^15^ In cell death processes mediated by protein degradation (e.g., via caspase proteases), a correlation between mRNA and protein levels cannot be presumed. Therefore, cell heterogeneity in death-related processes can best be studied with single-cell proteomics (SCP).

Compared to the rather well-developed single-cell transcriptomics approaches, SCP methods are still emerging.^16^ While some targeted antibody-based immunoassays have been applied to characterize proteins in single cells,^17,18^ these approaches are limited to a few dozen proteins per experiment and exhibit strong bias in quantification. Mass spectrometry (MS)-based proteomics can in principle overcome these limitations, but lacking the benefits of PCR, MS-based proteomic analysis at a single-cell level is very challenging due to (i) the extremely low amounts of proteins (ca. 0.2 ng in a mammalian cell), (ii) the high dynamic range of protein expression (7 orders of magnitude vs 3–4 orders for mRNAs),^19,20^ and (iii) the inevitable sample loss during protein extraction, digestion, and chromatographic separation of the peptide digest.^21^ Consequently, despite the introduction of ground-breaking SCP methods such as SCoPE-MS,^21^ SCoPE2^22,23^ and nanoPOTS,^24,25^ they have been able to analyze between 500 and 2000 proteins in diverse cell lines. Although a recent study has investigated the differentiation of monocytes to macrophage-like cells at the single-cell level upon chemical induction using phorbol 12-myristate 13-acetate,^23^ as cellular heterogeneity of differentiating stem cells in a leukemia culture model,^26^ SCP has so far not been able to apply the techniques of drug target identification, such as FITExP. Here, we demonstrate such an ability, thus pioneering single-cell chemical proteomics (SCCP).

Most SCP studies so far have considered two different types of cells (*e.g.*, monocytes *vs* macrophage cells or Jurkat vs U-937 cells)^21,23^ with vastly different proteomes. The separation of these cells by SCP was relatively straightforward as it could be done using a few most abundant proteins. In contrast, in cells influenced by a drug, the most significantly regulated proteins (drug targets) are seldom highly abundant, being frequently found in the abundance-sorted list below the 1000th position.^2^ Therefore, the SCCP development required achieving the following two intermediate objectives. First, average protein abundances in a homogeneous cell population measured by SCP must correlate with the abundances in bulk proteome analysis. This goal was achieved by starting from analyzing as bulk a relatively high number of cells and gradually reducing this number down to single cells, monitoring the correlation with the bulk analysis and systematically troubleshooting when this correlation broke down. A number of issues have been found and resolved related to protein extraction, digestion, labeling with isobaric reagents, LC separation, MS acquisition, and statistical analysis. In the end, satisfactory correlations between SCP and bulk proteomics results were consistently obtained. The second intermediate goal objective was to detect the known strong regulation of the drug targets as in FITExP, by SCP with high statistical significance. This again required systematic studies and optimizations.

**Figure 1:**
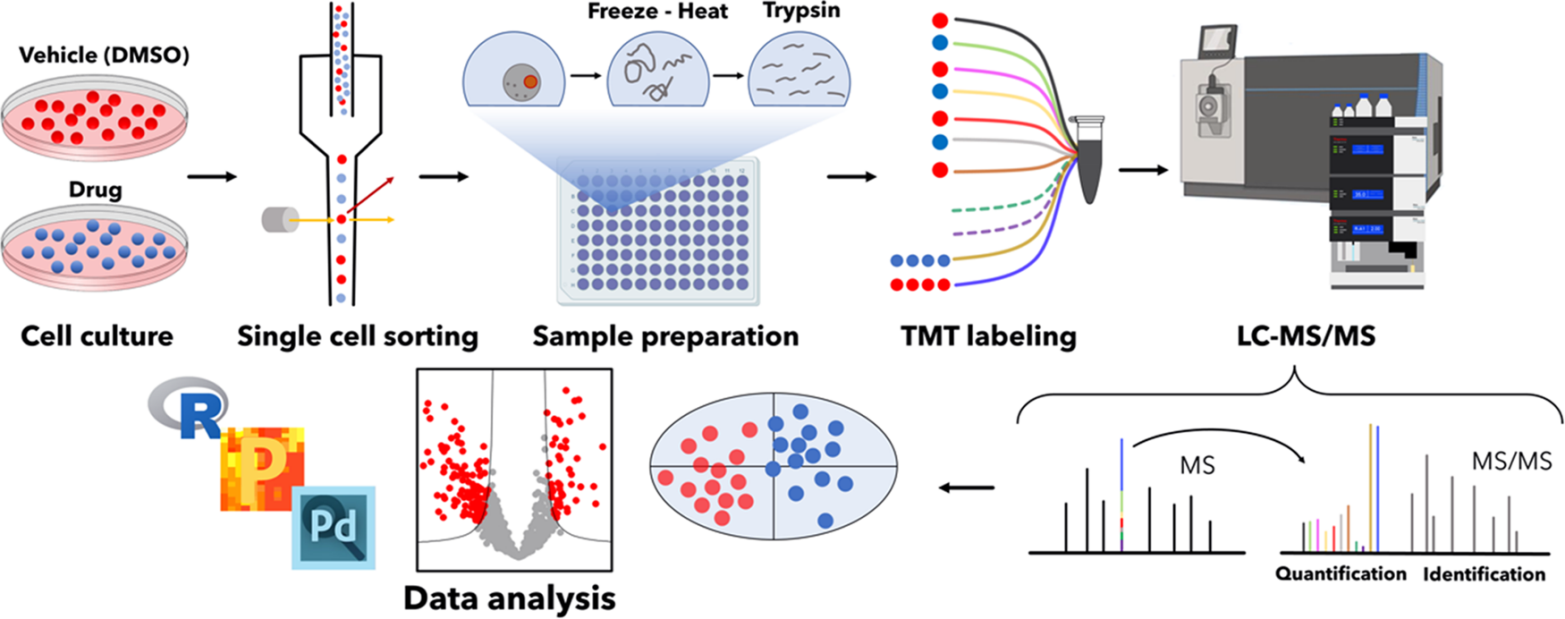
SCCP workflow. The workflow developed for SCCP included cell culturing and treatment with drugs, isolation of individual cells by FACS, protein extraction and digestion, TMT labeling of thus obtained tryptic peptides followed by multiplexing, LC-MS/MS, and statistical data analysis. All steps are optimized for achieving the desired proteome depth and quantitative correlation with bulk analysis. In the figure, the split carrier proteome occupies two channels (131N and 131C) in a TMT11plex set, with two other channels (130N and 130C) remaining empty (dotted lines). Identification of peptides is achieved via matching masses of sequence-specific fragments, and quantification is performed by the abundances of the low-mass TMT reporter ions.

Here, we present the SCCP workflow developed based on SCoPE-MS and applied to studying in a time-course manner the proteome effects of anticancer drugs MTX, camptothecin (CPT), and tomudex (TDX), also known as raltitrexed. These drugs were applied at LC_50_ concentration to A549 human lung adenocarcinoma cells, causing half of the cells to die within 48 h. Our workflow comprises the isolation of surviving cells using fluorescence-activated cell sorting (FACS), minimal sample preparation including tryptic digestion, tandem mass tag (TMT) isobaric labeling for protein quantification, incorporation of a carrier proteome (CP) to boost the MS signal,^27^ liquid chromatographic separation at a low flow rate, MS/MS data acquisitions, and SCCP-optimized data processing (Figure 1). The main goal of the study was to identify the time scale of the decision-making dying/surviving process, i.e., to reveal at what time the homogeneous cell population started to differentiate under the influence of a drug into cells committed to surviving or dying.

## Experimental Section

### Cell Culturing Treatment

Human A549 lung adenocarcinoma cells obtained from ATCC (Manassas, VA) were grown in Dulbecco’s modified Eagle’s medium (DMEM, Lonza, Walkersville, MD) supplemented with 10% fetal bovine serum (FBS) superior (Biochrom, Berlin, Germany), 2 mM l-glutamine (Lonza), and 100 U/mL penicillin/streptomycin (Thermo, Waltham, MA) at 37 °C in 5% CO_2_.

The LC_50_ values for the drugs (MTX, CPT, and TDX) were determined by the CellTiter-Blue cell viability assay (Promega), as described previously.^2^ Cells were seeded into 96 plates at a density of 3000 cells per well, and after 24 h of culture, they were treated with serial concentrations of the respective drug: MTX (0–100 μM), CPT (0–100 μM), and TDX (0–100 μM). After 48 h, the media were discarded and replaced with 100 μL of fresh culture media. In each well, 20 μL of resazurin (CellTiter-Blue Cell Viability Assay kit, Promega) was added to perform the viability assay. After 4 h of incubation at 37 °C, the fluorescence of wells was measured in an Infinite F200 Pro fluorometer (Tecan) by detecting the ratio between the excitation at 560 nm and emission at 590 nm. The LC_50_ values were determined from the dose–response curves by calculating the concentration causing the 50% fluorescence reduction compared with the untreated control (Figure S1).

Cells were then cultured and treated with MTX, CPT, and TDX at LC_50_ concentrations in 75 cm^2^ flasks for 3, 6, 12, 24, and 48 h (for CPT and TDX, only 12, 24, and 48 h treatments were performed). Control cells were treated with the vehicle, 10 mM dimethyl sulfoxide. At each incubation time point, the supernatant was collected and the attached surviving cells were disconnected from the surface with TrypLE (Gibco) for 5 min, after which they were harvested by centrifugation at 1000 rpm for 3 min. Detached dying cells were also collected after 48 h of treatment for bulk proteomics analysis. Both types of cells (detached and adhered) were washed twice with cold 1× phosphate-buffered saline.

### Isolation of Single Cells by FACS

For SCP analysis exclusively, the collected attached surviving cells were subjected to FACS analysis in FACSAria Fusion (BD Biosciences), in which cells were sorted based on the forward and side scatter (FSC/SSC) parameters only. Individual singlet cells were collected in a 96-well Lo-Bind plate (Eppendorf, Hamburg, Germany) containing 5 μL of 100 mM triethylammonium bicarbonate (TEAB) per well. A total of 96 single cells were sorted for each condition/drug (untreated and treated cells) using separate plates. In addition, a third plate was prepared, being dedicated only to CP, isolating 200 cells (100 treated and 100 control) per well in the first two rows. Altogether, 24 wells of CP cells were collected for each treatment and time point.

### Protein Extraction and Digestion

Proteins from single cells and CPs were extracted in four freeze–thaw cycles. Plates were frozen for 2 min in liquid nitrogen and immediately heated at 37 °C for 2 min. Proteins were denatured by heating the plates at 90 °C for 5 min. The resulted protein solutions were centrifuged at 1000 rpm for 2 min to spin down all of the volume present in the wells. Finally, 1 μL of 25 ng/μL sequencing grade trypsin (Promega, Madison, WA) in 100 mM TEAB was supplemented using a MANTIS automatic dispenser (Formulatrix, Bedford, MA). In the case of CP, digestion was achieved with addition of 2 μL trypsin solution. Plates were incubated at 37 °C overnight (ca. 16 h). Bulk proteomes of MTX-treated, control, and detached cells (about 500 cells per condition) after 48 h of treatment were prepared identically to CP samples and distributed to five TMT channels per condition in a TMTpro-labeled (15-plex) experiment.

### TMT Labeling

TMT10plex and TMT11plex including channel 131C were used in this study. Unless specified, each TMT10plex set contained four control cells and four treated cells with tags interspaced, as well as a single channel with CP (200 cells in channel 131).^28^ TMT 130N was not used to minimize the cross-contamination with the CP channel. Peptides were TMT-labeled by dispensing 1 μL of the respective TMT reagent dissolved in dry acetonitrile (ACN) at a concentration of 10 μg/μL using the MANTIS robot. Plates were incubated at room temperature (RT) for 2 h, and then, the reaction was quenched by adding 1 μL of 5% hydroxylamine (also with the automatic liquid handler), following incubation at RT for 15 min. In some experiments, the CP was split into two channels, 131N and 131C, one composed of 100 control cells and the other of 100 treated cells. Channels 130N and 130C were left empty to prevent cross-contamination from CP channels. The labeled samples were pooled together using a 10 μL glass syringe (VWR, Japan), starting always with the CP samples in each TMT set, to minimize sample loss during the pooling.^28^ Labeled peptides were pooled into MS sample vials with a glass insert (TPX snap ring vial from Genetec, Sweden) and dried in a speed vacuum concentrator (Concentrator Plus, Eppendorf). Dry peptides were resuspended in 7 μL of 2% ACN, 0.1% formic acid (FA) prior to LC-MS/MS analysis.

### RPLC-MS/MS Analysis

Peptide samples were separated on a Thermo Scientific Ultimate 3000 UHPLC (Thermo Fisher Scientific) using 10 min loading at a 3 μL/min flow rate to a trap column (Acclaim PepMap 100, 2 cm × 75 μm, 3 μm, 100 Å, Thermo Fisher Scientific). The separation was performed on an EASY-Spray C18 analytical column (25 cm × 75 μm, 1.9 μm, 300 Å, ES802A, Thermo Fisher Scientific). A constant flow rate of 100 nL/min was applied during sample separation achieved in a linear gradient ramped from 5% B to 27% B over 120 min, with solvents A and B being 2% ACN in 0.1% FA and 98% ACN in 0.1% FA, respectively. LC-MS/MS data were acquired on an Orbitrap Fusion Lumos Tribrid mass spectrometer (Thermo Fisher Scientific, San José CA), using nano-electrospray ionization in positive ion mode at a spray voltage of 1.9 kV. Data-dependent acquisition (DDA) mode parameters were set as follows: isolation of top 20 precursors in full mass spectra at 120 000 mass resolution in the *m/z* range of 375–1500, maximum allowed injection time of 100 ms, dynamic exclusion of 10 ppm for 45 s, MS2 isolation width of 0.7 Th with higher-energy collision dissociation (HCD) of 35% at a resolution of 50 000, and maximum injection time of 150 ms in a single microscan. The mass spectrometry proteomics data were deposited to the ProteomeXchange Consortium via the PRIDE partner repository^29^ with the dataset identifier PXD025481.

### Data Analysis

Raw data from LC-MS/MS were analyzed on Proteome Discoverer v2.4 (Thermo Fisher Scientific), searching proteins against the SwissProt human database (release July 30, 2019, with 20,373 entries) and known contaminants with Mascot Server v2.5.1 (MatrixScience Ltd., U.K.) allowing for up to two missed cleavages. Mass tolerance for the precursor and fragment ions was 10 ppm and 0.05 Da, respectively. Oxidation of methionine, deamidation of asparagine and glutamine as well as TMT adducts to lysine and N-termini were set as variable modifications. The percolator node^30^ in Proteome Discoverer was set to the target false discovery rate at 1% with validation based on the *q*-value.

The TMT reporter ion abundances (RIAs) at a peptide level were extracted from the search results. The subsequent analyses were performed in the RStudio (version 1.3.1073) programming language environment, the software for multivariate data analytics SIMCA (v. 15.0.2.5959, Sartorius), and the Perseus software platform.^31^ Peptides from single cells with RIAs exceeding 10% of the abundance values for the respective carrier channel were filtered out, being considered a result of co-isolation or other interferences, resulting in about 30% of the peptides discarded for further analysis (Figure S1A). After filtering, the remaining RIAs were arranged into a matrix of peptide IDs (rows) vs single cells (columns). All RIAs were log2-transformed, and the data were normalized in columns by subtracting their median values computed, ignoring the missing values. Peptides quantified in less than 10 cells were discarded (usually <0.05% peptides per dataset). Protein-level quantification was achieved by attributing each unique peptide to its respective top-ranked protein within a protein group. As protein relative abundance, the median RIA value among the peptides belonging to that protein was taken. The new relative abundance matrix (protein IDs vs single cells) was again normalized by calculating the median value for each column (or single cell) and then subtracting the median value calculated for each abundance on the respective column. Missing values in the resulting matrix were imputed based on the normal distribution of valid values (method available in the Perseus software platform^32^), using a width of 0.3 standard deviations of the Gaussian distribution of the valid values and a downshift of 1.8 standard deviations. Finally, the batch effects across the TMT sets were corrected by applying an empirical Bayesian framework in the SVA package^33^ (for the schematic workflow of the data analysis and an example for batch correction) (Figure S1B,C).

The obtained matrix of relative protein abundances was used for statistical analysis. Principal component analysis (PCA) was performed to determine the separation degree between the control and drug-treated cells and to identify the outliers (single cells outside the limits of the PCA diagram with *p* < 0.05), which were removed from subsequent analysis. The resulting data were analyzed by OPLS-DA and clustering analysis, and the fold changes were presented as volcano plots.

## Results and Discussion

### Time-Course Single-Cell Chemical Proteomics Analysis

**Figure 2:**
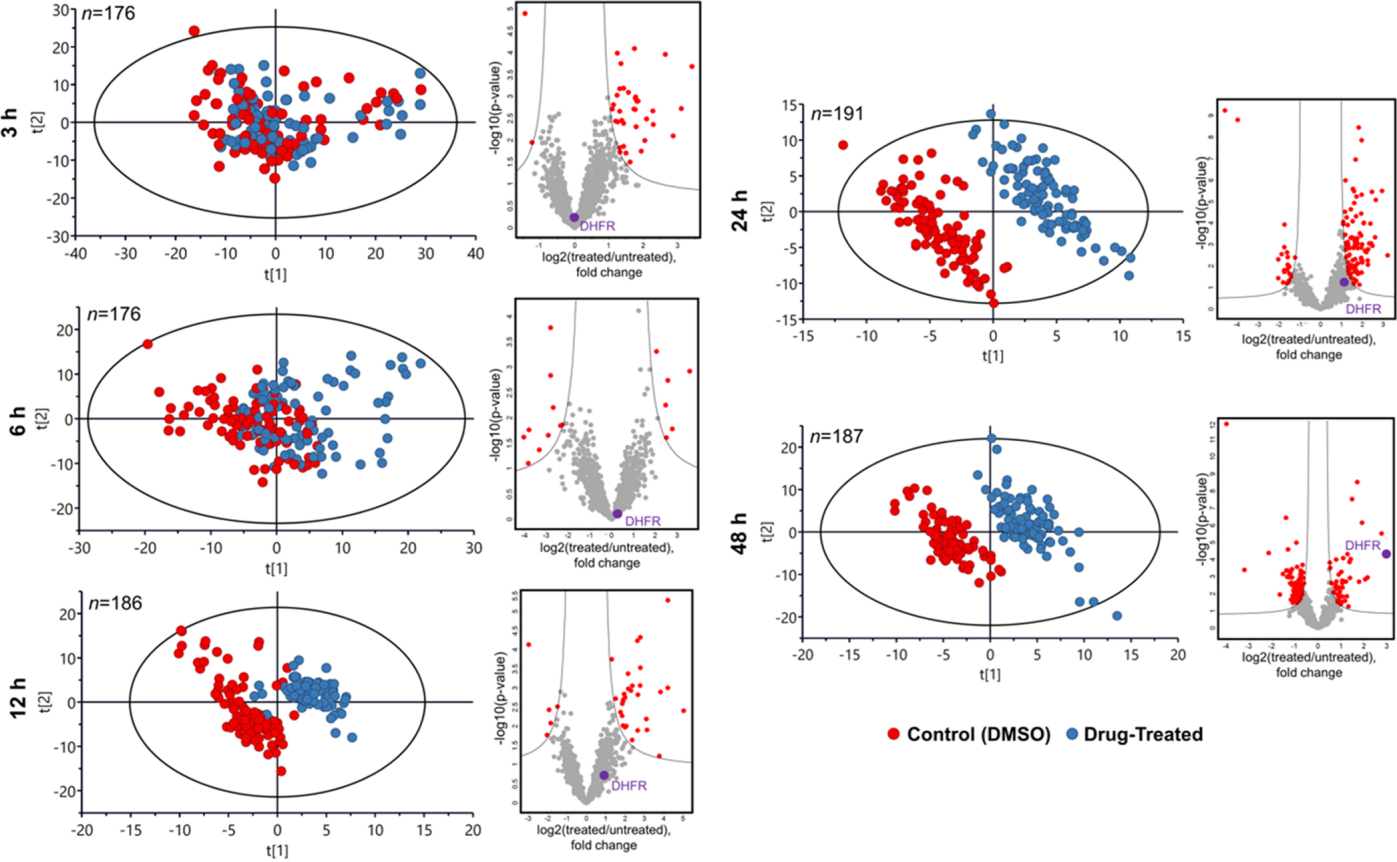
Time-course results upon treatment with methotrexate. PCA plots of single-cell data as a time course demonstrating the emergence of separation between the MTX-treated and untreated attached cells with incubation time, and the corresponding volcano plots of regulated proteins showing the emergence of dihydrofolate reductase (as indicated with a purple dot and DHFR) among the top regulated proteins.

The goal of the experiment was to determine the time point at which an attached cell makes the decision to die so that its proteome becomes altered to resemble that of the end-point-detached (dying) cells rather than the end-point-attached (surviving) cells. For that purpose, the cells were treated with MTX for 3, 6, 12, 24, and 48 h at an LC_50_ concentration of 1.15 μM (Figure S2). The attached cells at each time point and the detached cells at 48 h were collected. The FACS-isolated (Figure S3) 96 treated and 96 control cells were analyzed by SCCP at each time point, using a CP representing a mixture of 100 treated and 100 untreated attached cells. The bulk proteomes of 48 h detached and attached treated cells were analyzed separately. On average, over 1500 proteins and 10 000 peptides were identified and quantified in single cells at each incubation time. Figure 2 shows how the attached treated and untreated cell populations, being almost indistinguishable on a PCA plot at 3 h treatment, become gradually separated with time, achieving nearly full separation at 12 h. The separation is driven by the alterations induced in the proteome, as no batch correlation (Figure S4) and only minor TMT reagent-related grouping in the early time points was observed (Figure S5).

**Figure 3:**
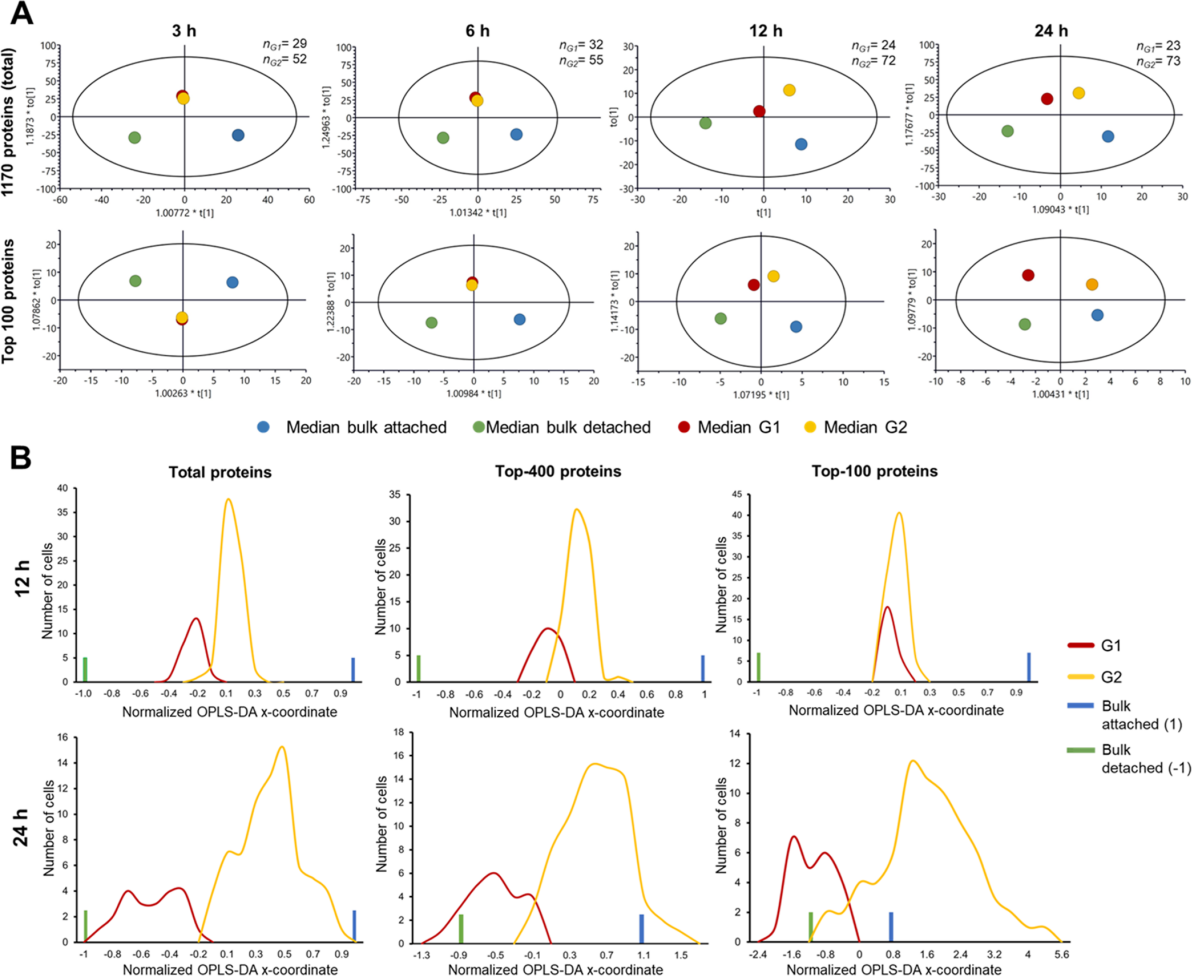
Statistical analysis of single cells treated with methotrexate. (A) OPLS-DA analysis of SCCP data on median protein abundances in G1 and G2 cell groups from MTX-treated single cells at different time points together with bulk abundances for the total proteome (1170 proteins) and top 100 most abundant proteins. The numbers of single cells belonging to G1 and G2 are given at the right top of each plot. (B) Distribution of the main OPLS-DA coordinates of G1 and G2 groups of MTX-treated attached cells at 12 and 24 h past treatment for the total proteome, top 400, and top 100 proteins. The *x*-coordinates were normalized such that the coordinates of the attached and detached cells’ bulk-analyzed proteomes after 48 h treatment are +1 and −1, respectively.

A hierarchical cluster analysis of SCP abundances was performed to identify the cell subgroups in the treated attached population of surviving cells that were committed either to death or to survival at each time point (Figure S6). It was assumed that the two most abundant cell clusters represent the subgroups of the future dying (G1) and surviving cells (G2). The hypothesis was that being put on a PCA plot together with the 48 h attached and detached cells in bulk analysis representing the two ultimate cell destinies, the two subgroups will reveal their identities by being closer to the respective destiny type (Figure 3A). For time points earlier than the commitment event, cell clustering into the two subgroups will be random (Figure S7), and thus, both subgroups of single treated cells would end up in the middle of the orthogonal partial least-squares discriminant analysis (OPLS-DA) plot close to each other.

Both these predictions were confirmed when the median abundances of all 1170 quantified proteins and 100 most abundant proteins in G1- and G2-treated single cells separated by clustering analysis of attached cells were used for building an OPLS-DA model. The model also included the data on 48 h attached cells and 48 h detached cells, which represented the final destinations of the survival and dying subpopulations (Figure 3A). For 3 and 6 h treatments, there was an overlap of the G1 and G2 clusters, whereas a definite separation between them in the direction of the destiny points was obtained at 12 h and longer treatment times. Single cells in G1 were thus acquiring a proteome profile corresponding to the dying fate, while single cells in G2 represented the surviving subpopulation. As expected, the OPLS-DA separation between these two subpopulations of treated single cells grew with time. Similarly, the number of proteins with significantly changed abundances between the vehicle- and MTX-treated populations increased with time from 32 and 15 proteins at 3 and 6 h to 38, 121, and 134 proteins at 12, 24, and 48 h, respectively. These significantly regulated proteins were found with a wide range of abundances (Figures S8 and S9). Therefore, the A549 cell commitment to death occurs between 6 and 12 h past MTX treatment (Figure S10). This time scale is consistent with the earlier reports on dynamic proteomics measurements in cells treated with a drug at LC_50_; in the first hours past treatment, the cells try to overcome the encountered difficulty, activating survival pathways, and only commit to death after such an attempt fails.^1,34^

Interestingly, at 12 h, more separation was seen for the whole proteome, while at 24 h, the 100 most abundant proteins showed bigger separation. This observation agreed well with the notion that the cell path to death starts with the inner mechanism altering lower-abundant mechanistic proteins first, followed by the altering household proteins that change the cell morphology. Consistent with this scenario, when the main OPLS-DA coordinates of individual cells were plotted on a scale normalized such that the attached cells treated for 48 h had *x* = 1 and the corresponding detached cells had *x* = −1 (as determined in bulk analyses), the obtained distributions of G1 and G2 single cells were separated in 12 h for the full proteome, but less so for 400 most abundant proteins and not at all for top 100 proteins (Figure 3B). At the same time, for 24 h treatment, the G1 and G2 proteomes gave broad distributions separated more for highly abundant proteins, suggesting that cell morphology alteration is well underway.

Pathway analysis of 179 proteins with significantly different abundances in G1 vs G2 of treated single cells at 12 h past MTX treatment revealed that they preferentially belong to metabolic, carbon metabolism, and ribosome- and proteasome-related pathways (Figure S11 and Data S1). More specifically, the G1 subgroup is enriched in proteins involved in ribosome- and proteasome-related pathways, meanwhile the G2 subgroup is enriched in metabolic pathways.

### SCCP with Camptothecin and Tomudex (Raltitrexed)

**Figure 4:**
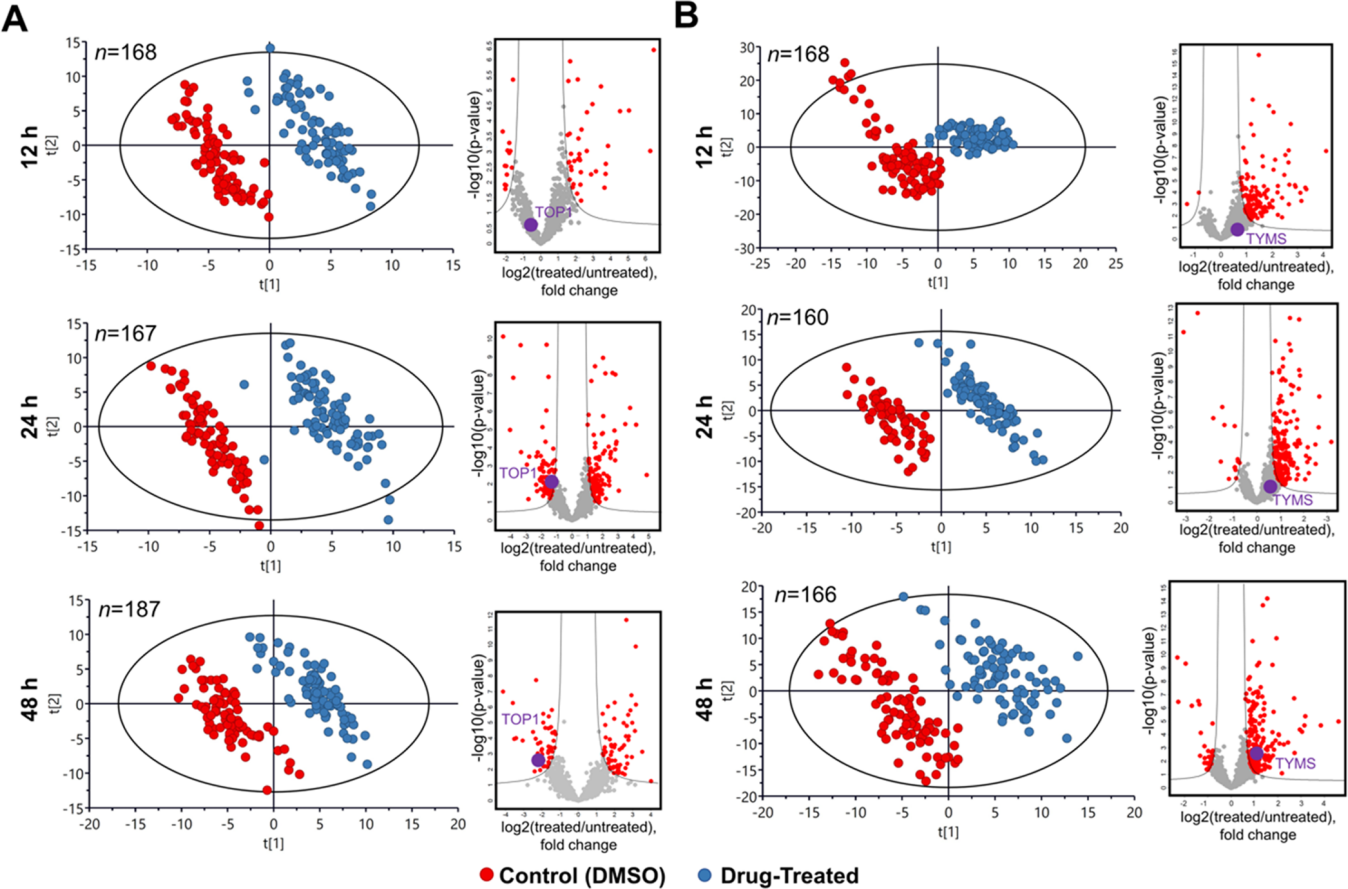
Time-course results upon treatment with camptothecin and tomudex. PCA plots of SCCP data as a time course demonstrating the emergence separation between the untreated cells and the attached cells treated with (A) camptothecin and (B) tomudex with incubation time and the corresponding volcano plots of regulated proteins (as indicated with a purple dot and TOP1 or TYMS, respectively) showing the emergence of the known drug target among the top regulated proteins.

Similar results to MTX were obtained in single A549 cells treated with CPT (LC_50_ = 3 μM) and TDX (LC_50_ = 50 μM). Their known targets, downregulated DNA topoisomerase 1 (TOP1) and upregulated thymidylate synthetase (TYMS), emerged among the top proteins in the respective areas of the volcano plot (Figure 4). While these drugs have different mechanisms of actions and targets, the A549 cells have clearly formed two well-separated clusters in PCA.

### Comparison of SCP with Diluted Bulk Proteomes

In an attempt to benchmark SCP depth of analysis, we have investigated the proteome profiles of diluted bulk samples of MTX-treated and untreated cells. Surprisingly, we found that the target protein (DHFR) was not detected in diluted samples when injecting 40 ng of the total protein amount into a column, which approximately represents an equivalent of a single-cell analysis. At the same time, DHFR was detected and quantified in most cells in SCCP analysis. We rationalized this puzzling result as follows. As the protein level in a bulk sample reflects the average level of that protein expression in single cells, half of the single cells express those proteins at higher levels than the bulk levels. Therefore, this protein can be detected in many single cells while not being detected in a bulk sample. Also, dilution analysis is typically performed in a few (3–5) replicates, while single cells are analyzed in 50–100 cells per group, which due to the statistical nature of data-dependent acquisition increases the detection probability of a low-abundant protein in at least several cells. Overall, this reasoning supports a higher detection probability of proteins in SCP compared to bulk proteomics.

### Target Percolation by OPLS-DA of Drug-Treated Single Cells

**Figure 5:**
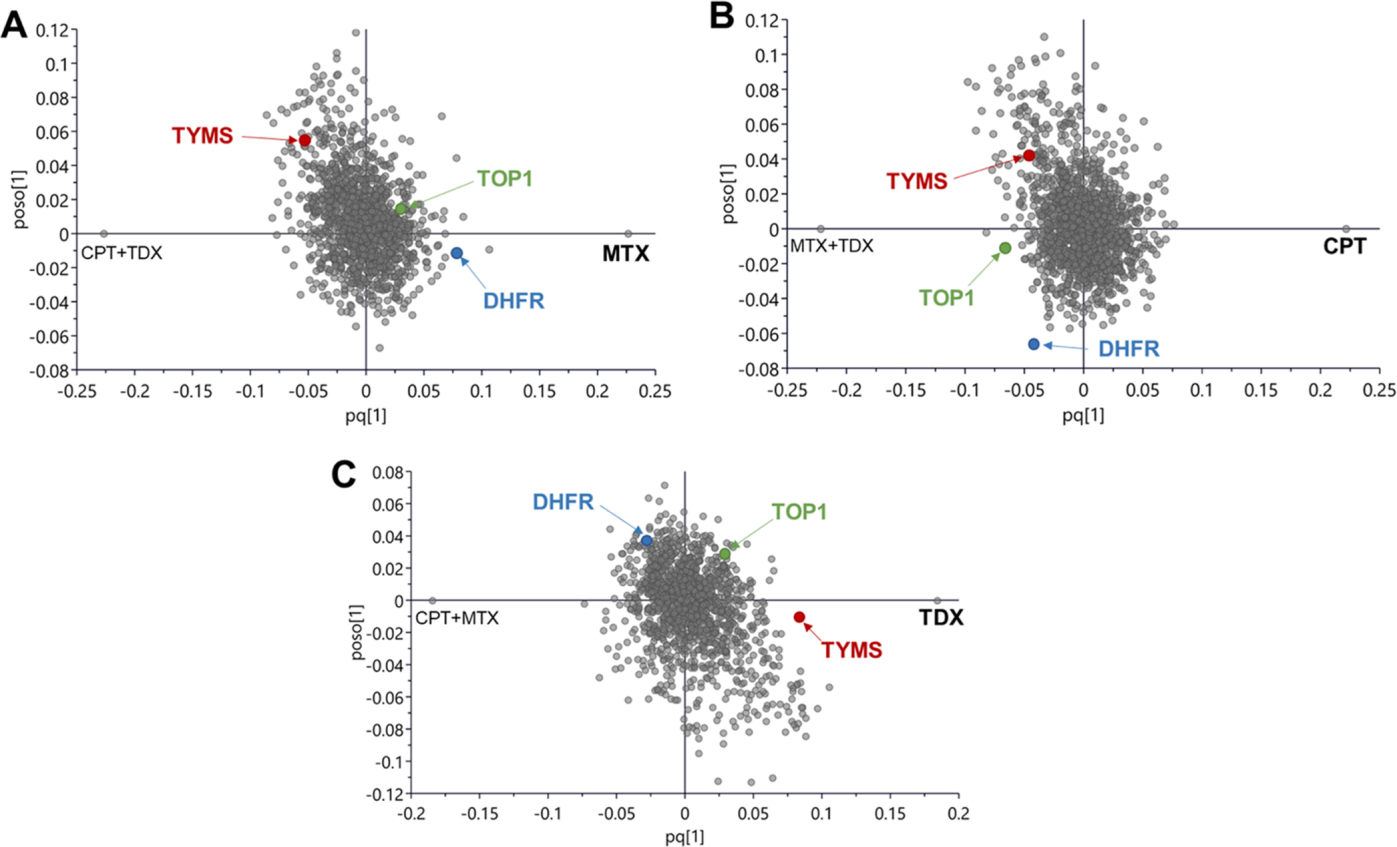
OPLS-DA analysis. OPLS-DA analysis contrasting the effect of one drug, (A) methotrexate, (B) camptothecin, and (C) tomudex, against the other two drugs, indicating the positions of their target proteins, DHFR, TOP1, and TYMS, respectively.

The ultimate goal of a chemical proteomics experiment is drug target identification, which can be obtained by contrasting a specific treatment against all other treatments and controls. While designing ProTargetMiner,^2^ we found that on average it takes 30–50 contrasting treatments to identify (“percolate”) the target uniquely among thousands of proteins in the proteome as the most specifically up- or downregulated protein. Here, we merged the MTX, CPT, and TDX SCCP data (treatment vs untreated control) at 48 h of treatment and contrasted one drug against the other two (Figure 5). For MTX, the target dihydrofolate reductase was the 4^th^ most specifically upregulated protein; whereas for CPT, TOP1 was the 15th most specifically downregulated protein; and for TDX, TYMS was the 10th most specifically upregulated protein. These results demonstrate that SCCP has the potential for unique drug target identification, provided enough contrasting treatments are obtained.

Considering that cell-to-cell heterogeneity is a fundamental property of highly complex cellular systems,^18^ the analysis of proteomes at a single-cell level is essential for understanding the complex diseases, such as cancer, where diverse phenotypes contribute to the survival and progression,^35^ as well as for studying the mechanisms of cell resistance to anticancer treatment.

Here, we demonstrated that SCP can be sufficiently quantitative for enabling chemical proteomics approaches for drug target identification and monitoring. The ability to “percolate” the probable drug target candidates by a contrasting OPLS-DA analysis has a paramount importance for the use of such a powerful drug target deconvolution method as ProTargetMiner.^2^

## Conclusions

The most important finding was that the SCCP time-course analysis provided new biologically relevant information, confirming that cell commitment to death can now be studied at a proteome level for individual cells. Between 6 h and 12 h past treatment, a large group of attached surviving drug-treated individual cells already committed to death (to be detached) formed a floating population that can only be recognized by SCP analysis. Notably, these changes were detected among the lower-abundant proteins, while highly abundant proteins remained at that point unaffected. Furthermore, it was even possible to determine the pathways and parts of cell machinery participating in the decision-making process.

After the quantitative aspect of single-cell proteomics has been improved, chemical proteomics at the level of single cells became reality. The technical innovation of the split carrier proteome (100–100 control and treated cells in two TMT channels) enabled monitoring of protein regulations at a bulk-like (“semi-bulk”) level and improved correlation between the bulk proteome and single-cell proteome data. The detailed profiling with SCCP of the heterogeneity of cancer cell response to drugs or treatments and the mechanistic analysis with a cellular resolution of resistance to therapy is now possible. Moreover, the FITExP method of chemical proteomics is now applicable to single cells. Like many novel analytical approaches, SCP is currently searching for the breakthrough application that alone could justify this method and possess the capacity to dominate the applications. Exploring the heterogeneous response to drugs of cancer cells by SCCP might prove to be such an application for single-cell proteomics.

The remaining challenges are however vast. For example, SCCP needs to provide deeper proteome analysis, targeting the benchmark of 5000 proteins quantified with ≥2 peptides. A great achievement would be if complementary tools of chemical proteomics, such as the proteome-wide integral solubility alteration (PISA) assay,^36^ could be implemented for single cells. With PISA, one could monitor the protein target engagement of the drug molecule. This, however, requires significant efforts in improving the methods of handling and analyzing ultrasmall protein amounts.

## Supporting Information Available

Supplementary figures (Figures S1–S11) (PDF)
Processed mass spectrometric data used for analysis and interpretation (XLSX)
