# Supplementary material for: Single-Cell Chemical Proteomics (SCCP) Interrogates the Timing and Heterogeneity of Cancer Cell Commitment to Death
Source: Anal Chem. 2022 Jun 22;94(26):9261–9. doi: 10.1021/acs.analchem.2c00413 (PMC9260713; doi:10.1021/acs.analchem.2c00413)
Supplement: Supplementary file 1 — ac2c00413_si_001.pdf [file ac2c00413_si_001.pdf]

Supporting Information

Single Cell Chemical Proteomics (SCCP) Interrogates the Timing and Heterogeneity of Cancer Cell Commitment to Death

Ákos Végvári*, Jimmy E Rodriguez, Roman A Zubarev*

Division of Chemistry I, Department of Medical Biochemistry & Biophysics, Karolinska Institutet, Biomedicum A9, Solnavägen 9, SE-171 77 Stockholm, Sweden

*Corresponding authors: Ákos Végvári (akos.vegvari@ki.se) and Roman A Zubarev (roman.zubarev@ki.se)

Table of contents:

Supplementary figures ____ *S2*

Figure S1. ____ **S2**

Figure S2. ____ **S4**

Figure S3. ____ **S5**

Figure S4. ____ **S5**

Figure S5. ____ **S8**

Figure S6. ____ **S10**

Figure S7. ____ **S11**

Figure S8. ____ **S14**

Figure S9. ____ **S17**

Figure S10. ____ **S23**

Figure S11. ____ **S24**

Supplementary figures

Figure S1. A) Summary table with the number of peptides and proteins identified in the raw data before filtering and after applying the data analysis workflow. B) Schematic representation of the bioinformatic workflow applied to the data acquired with single cells. C) As an example, PCA of eight TMT-sets are depicted before and after the batch correction.

A

	Before filtering		After filtering	
	Peptide	Protein	Peptide	Protein
MTX_3h	17757	2325	12910	1931
MTX_6h	13731	1988	9767	1625
MTX_12h	20415	2481	19405	1585
MTX_24h	17874	2240	17074	1627
MTX_48h	19664	2397	11692	1738
CPT_12h	18055	2188	9945	1487
CPT_24h	16099	1958	10802	1570
CPT_48h	17710	2244	10940	1662
TDX_12h	17744	2169	12808	1786
TDX_24h	15790	2037	15212	1746
TDX_48h	16464	2132	12076	1833

B

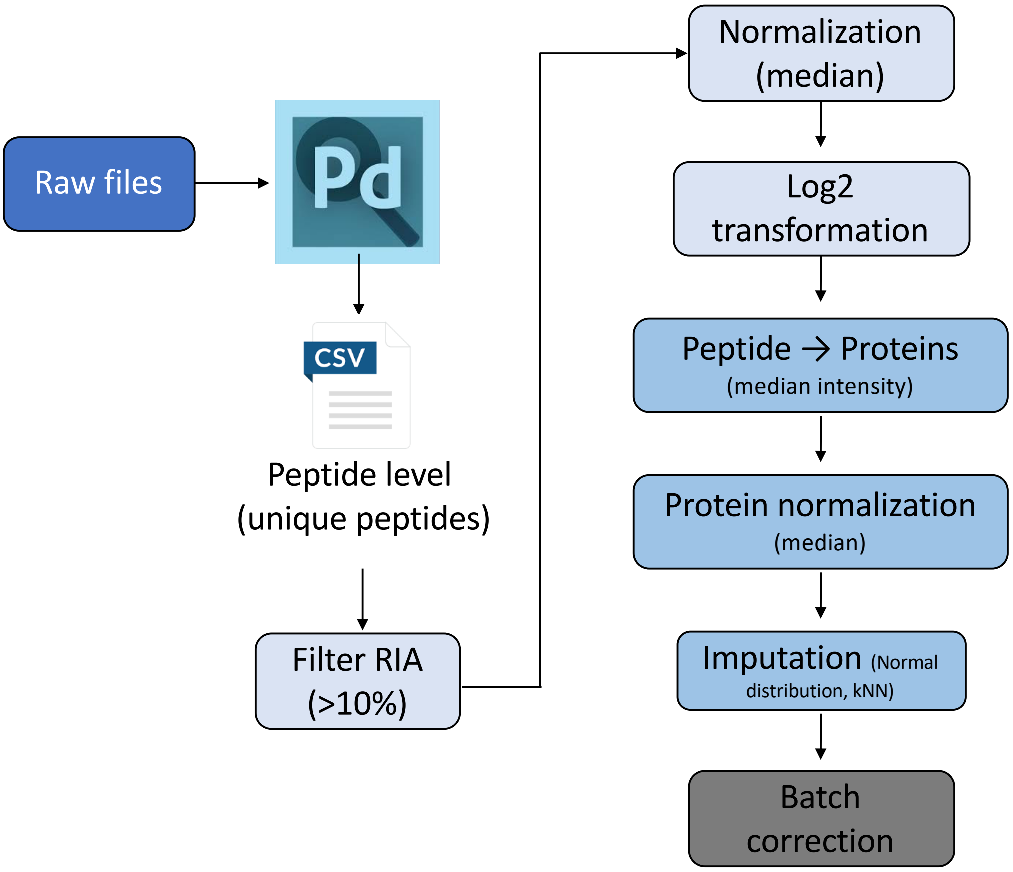

C

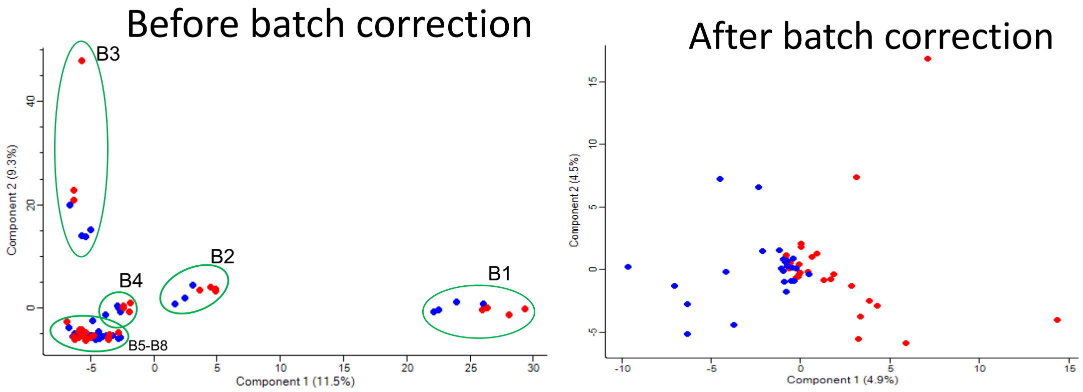

Before batch correction
After batch correction
B3
B4
B2
B1
B5-B8
Component 2 (9.3%)
Component 1 (11.5%)
Component 2 (4.5%)
Component 1 (4.9%)

Figure S2. Cell viability diagrams for three drugs (methotrexate, MTX; camptothecin, CPT; tomudex, TDX) used with A549 cells.

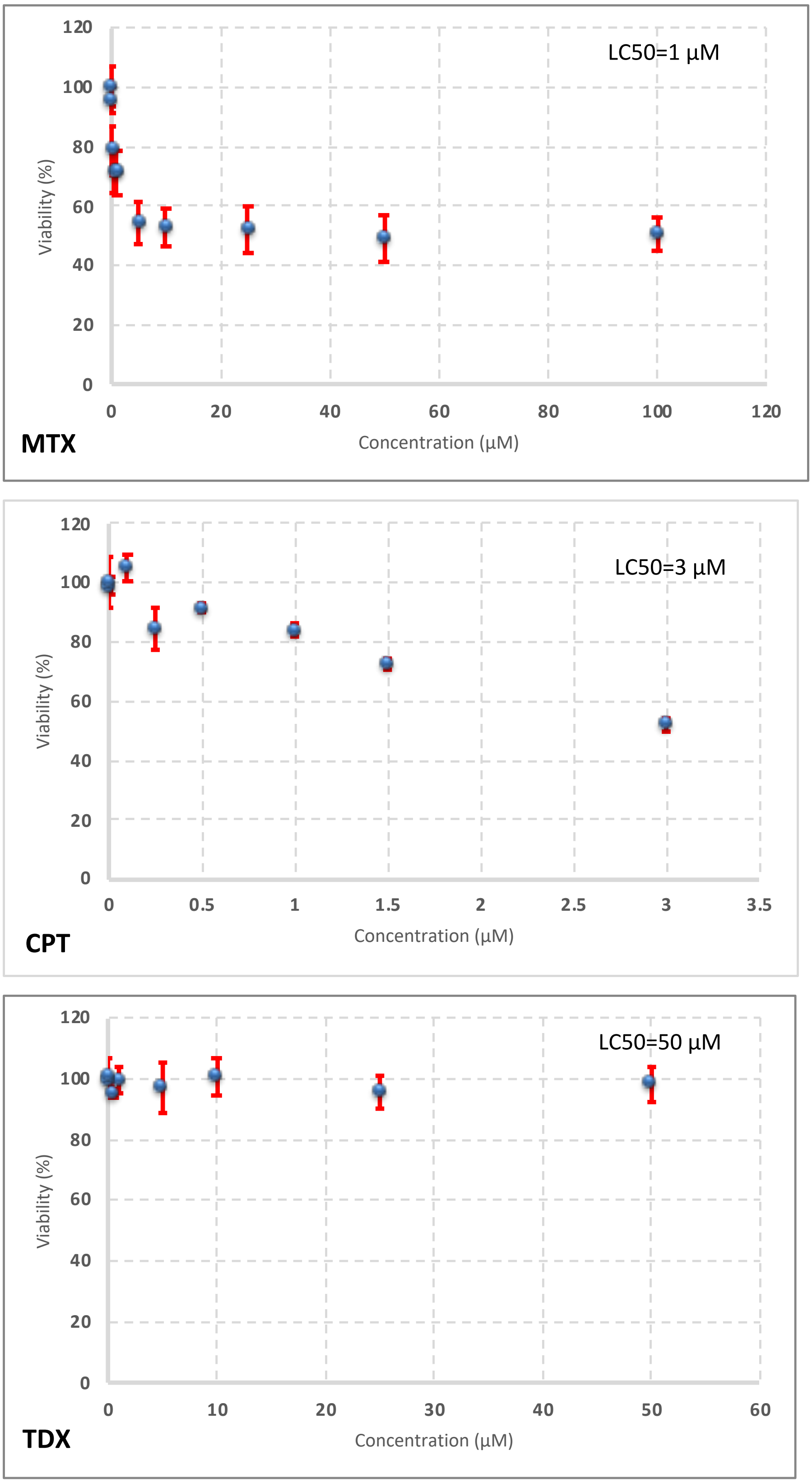

Figure S3. FACS diagrams with gating conditions for isolating individual DMSO-treated (A) and MTX-treated (B) A549 cells after 48 h.

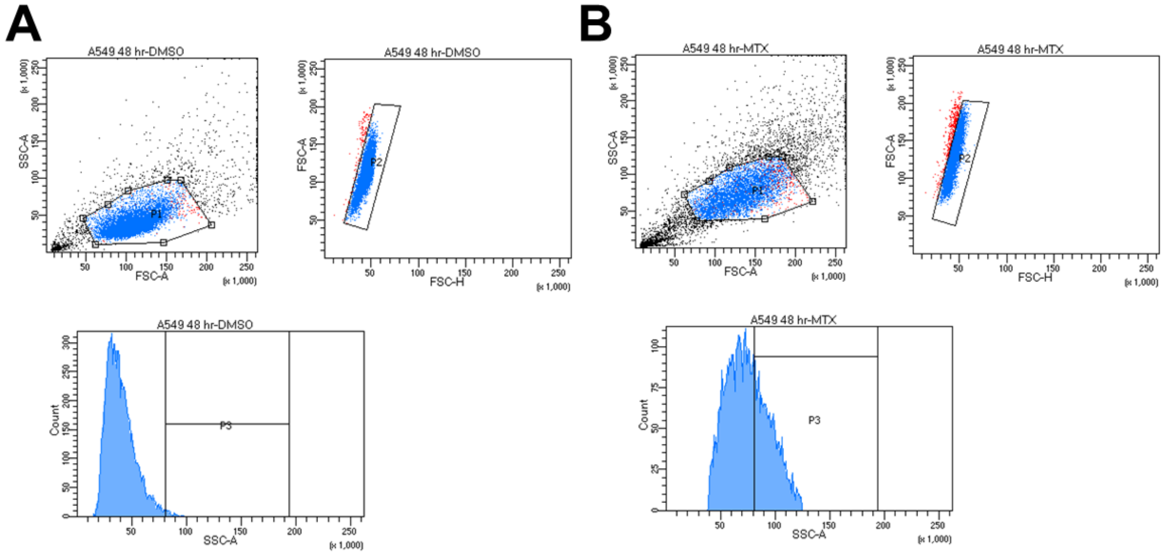

Figure S4. PCA plots of the proteomics results on MTX treated single cell presented in *Figure 2*, coloring the cells after the batches.

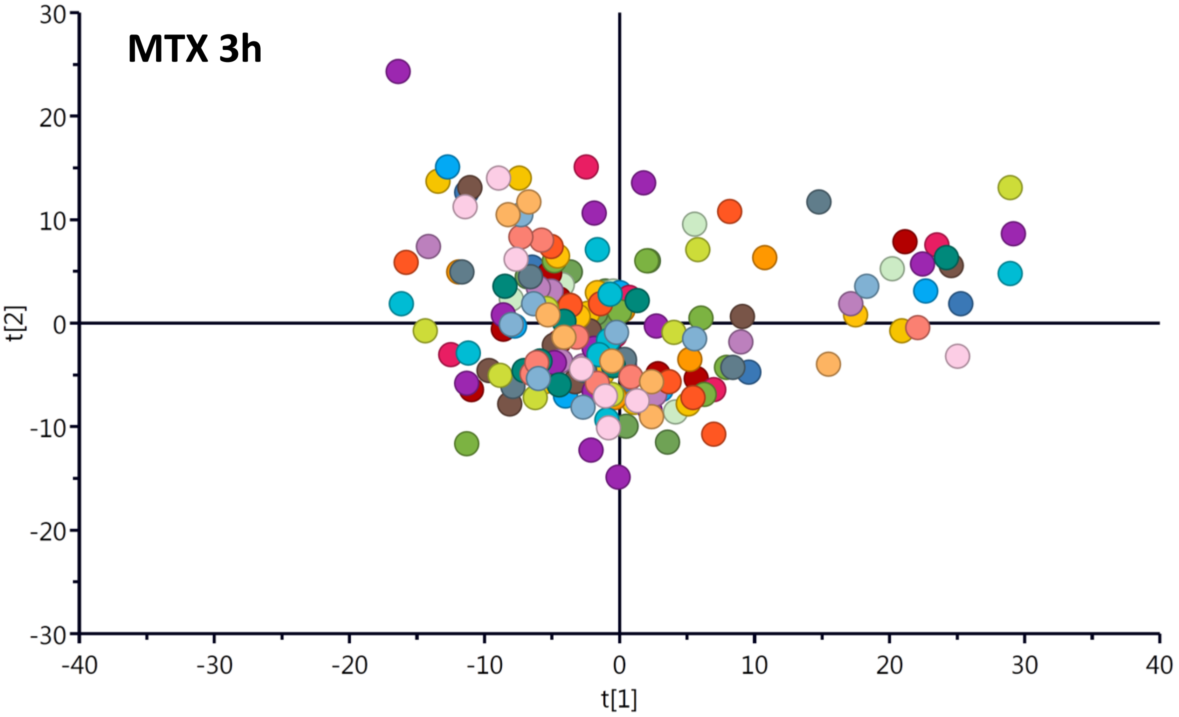

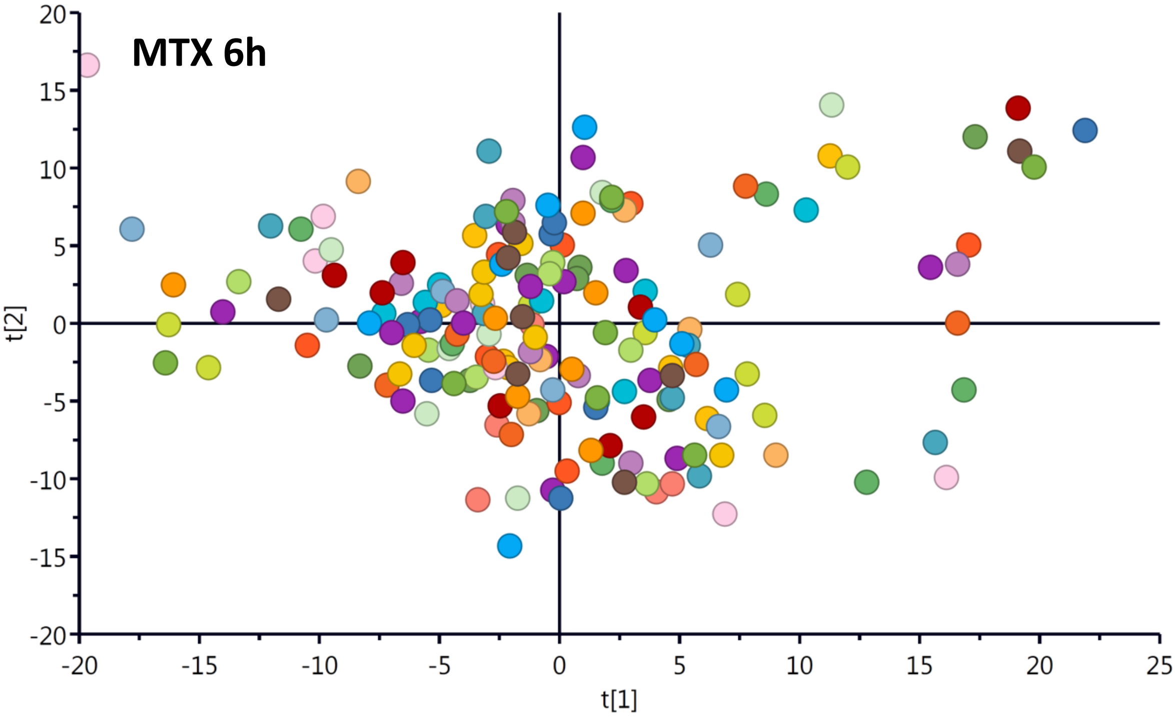
MTX 6h
t[2]
t[1]
20
15
10
5
0
-5
-10
-15
-20
-20
-15
-10
-5
0
5
10
15
20
25

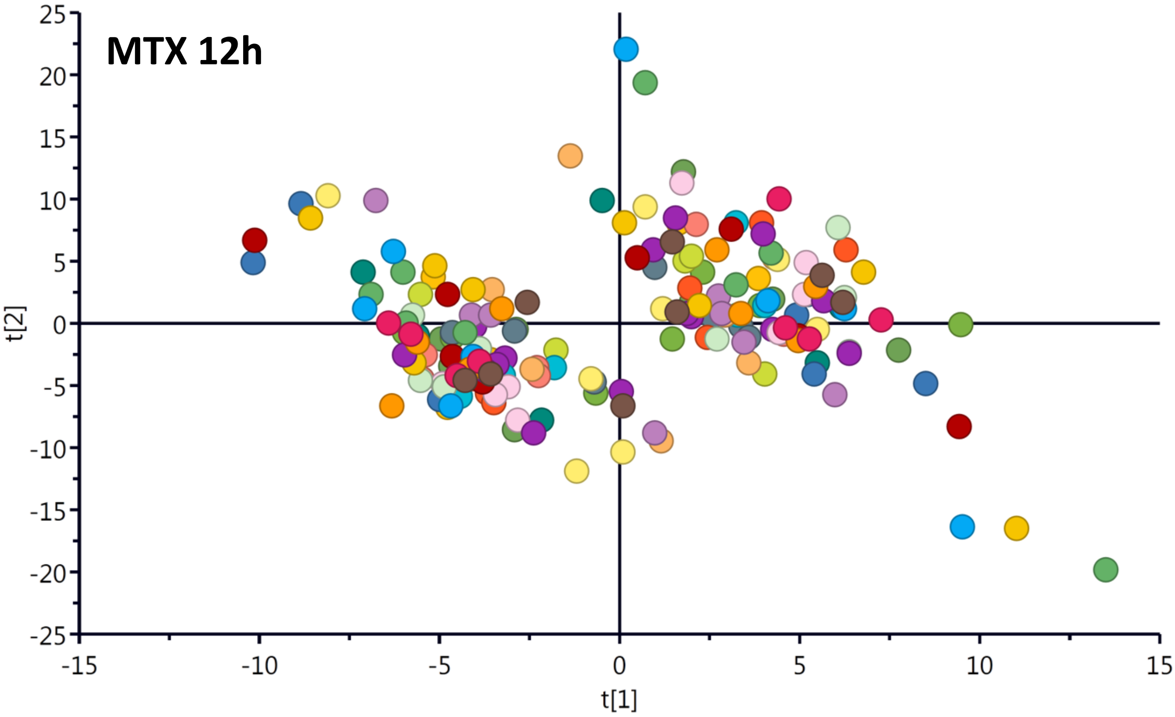
MTX 12h
t[2]
t[1]
25
20
15
10
5
0
-5
-10
-15
-20
-25
-15
-10
-5
0
5
10
15

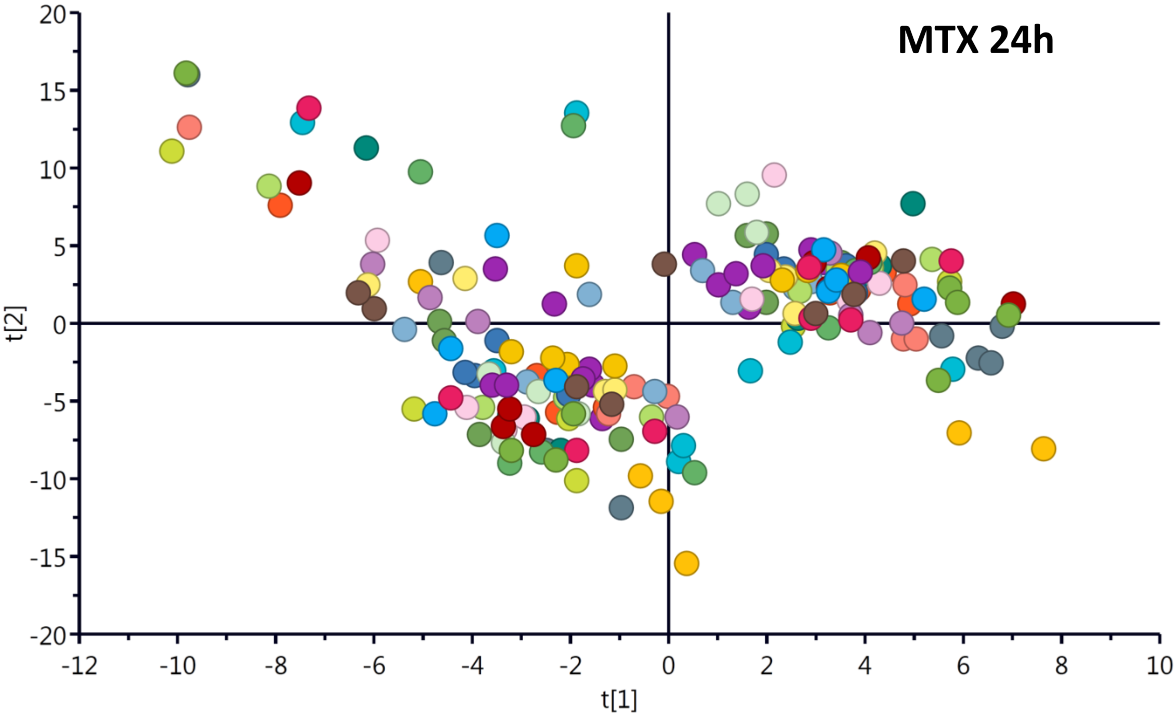
MTX 24h
t[2]
t[1]
20
15
10
5
0
-5
-10
-15
-20
-12
-10
-8
-6
-4
-2
0
2
4
6
8
10

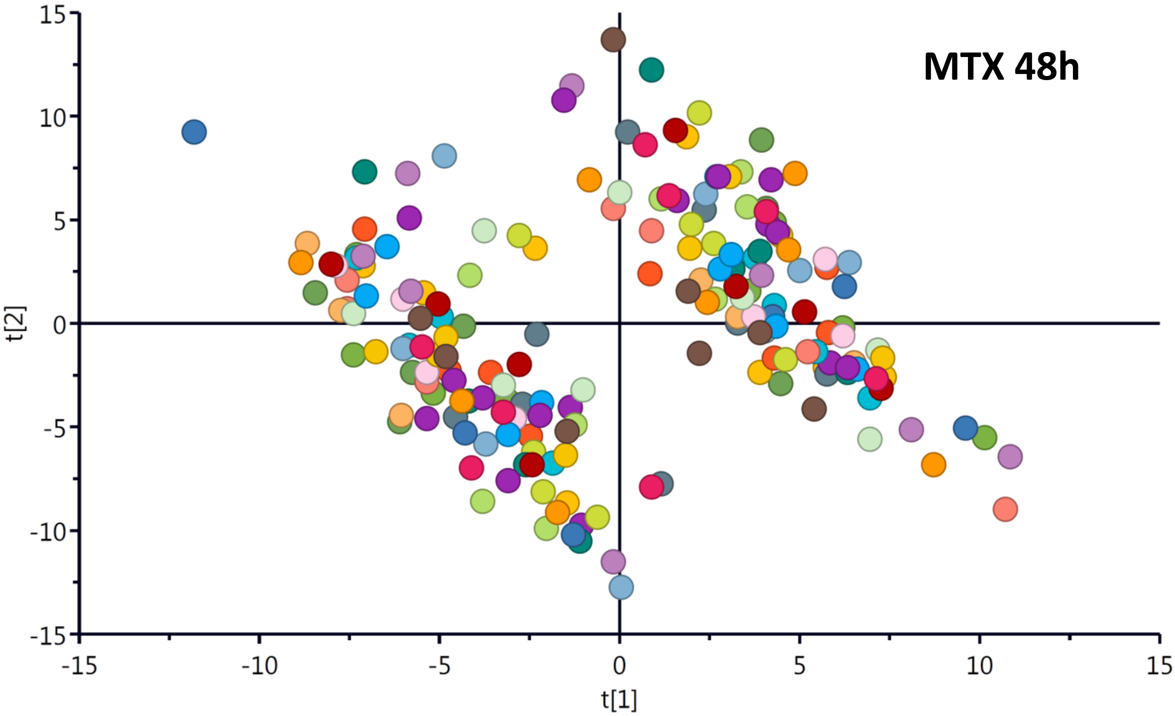
MTX 48h
t[2]
t[1]
15
10
5
0
-5
-10
-15
-15
-10
-5
0
5
10
15

Figure S5. Same as in Figure S4, coloring the cells after the TMT channels.

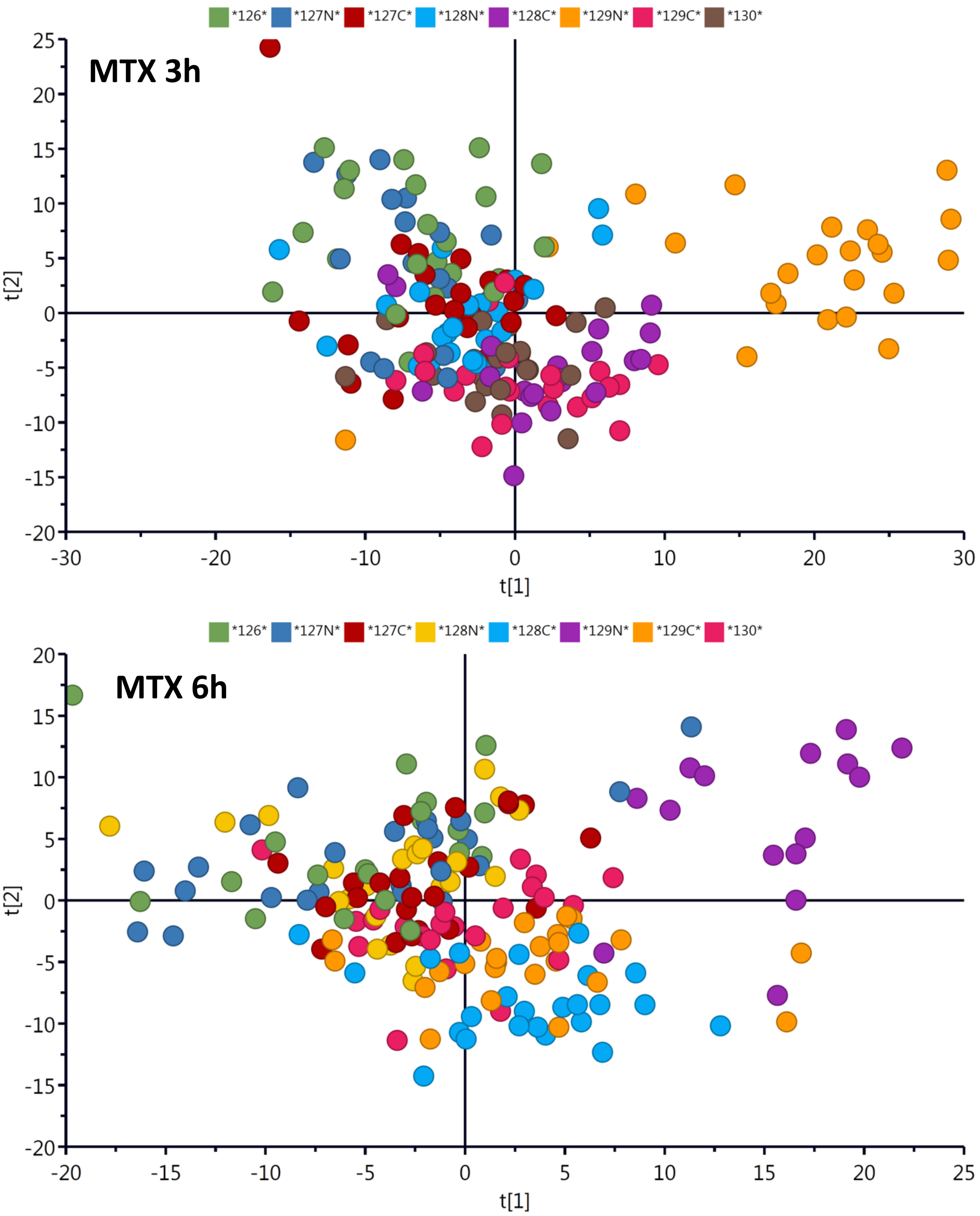

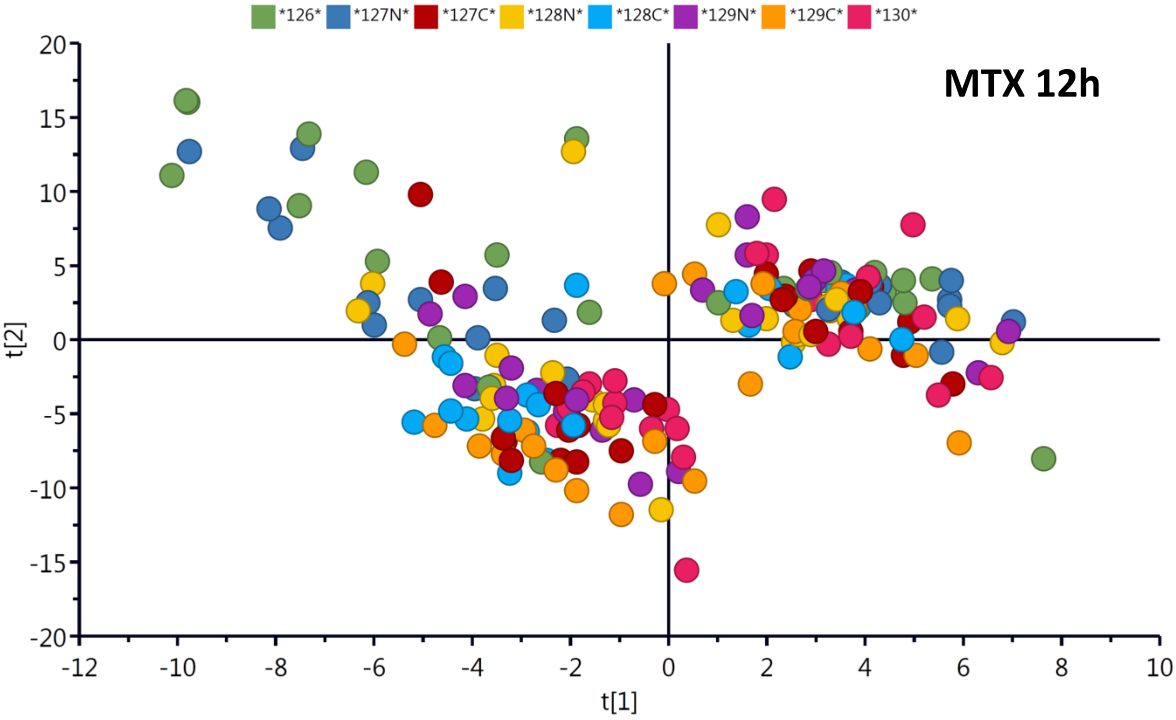
126 *127N* *127C* *128N* *128C* *129N* *129C* *130*
MTX 12h
t[2]
t[1]

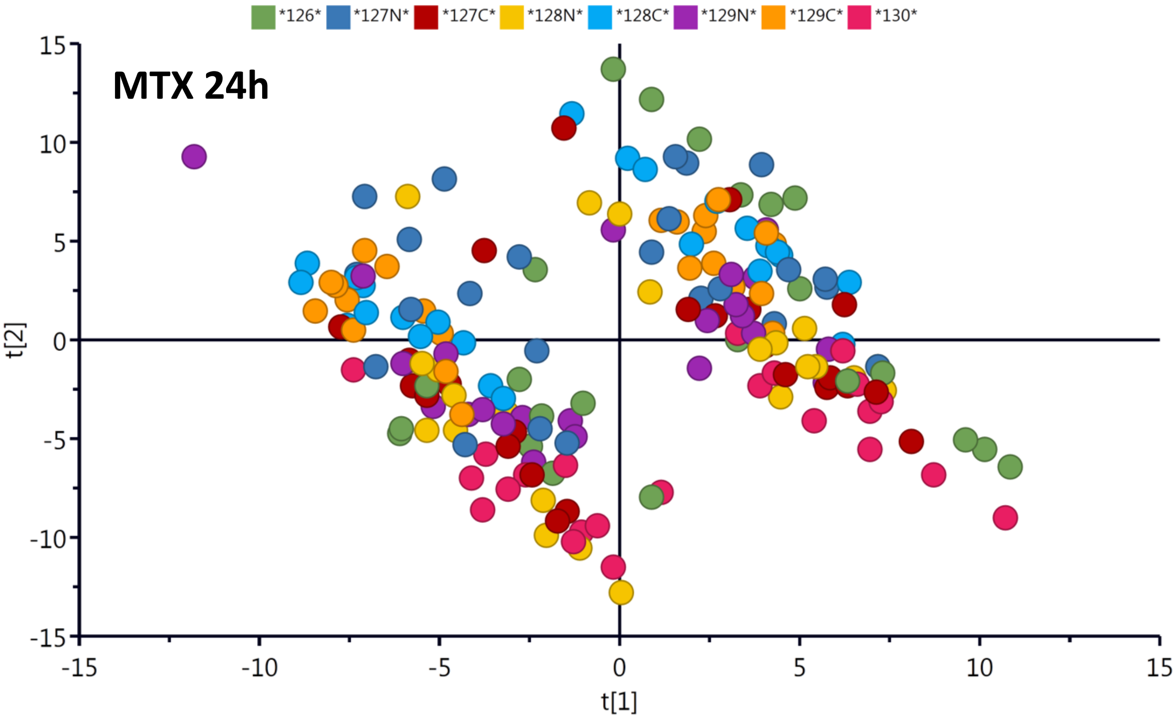
126 *127N* *127C* *128N* *128C* *129N* *129C* *130*
MTX 24h
t[2]
t[1]

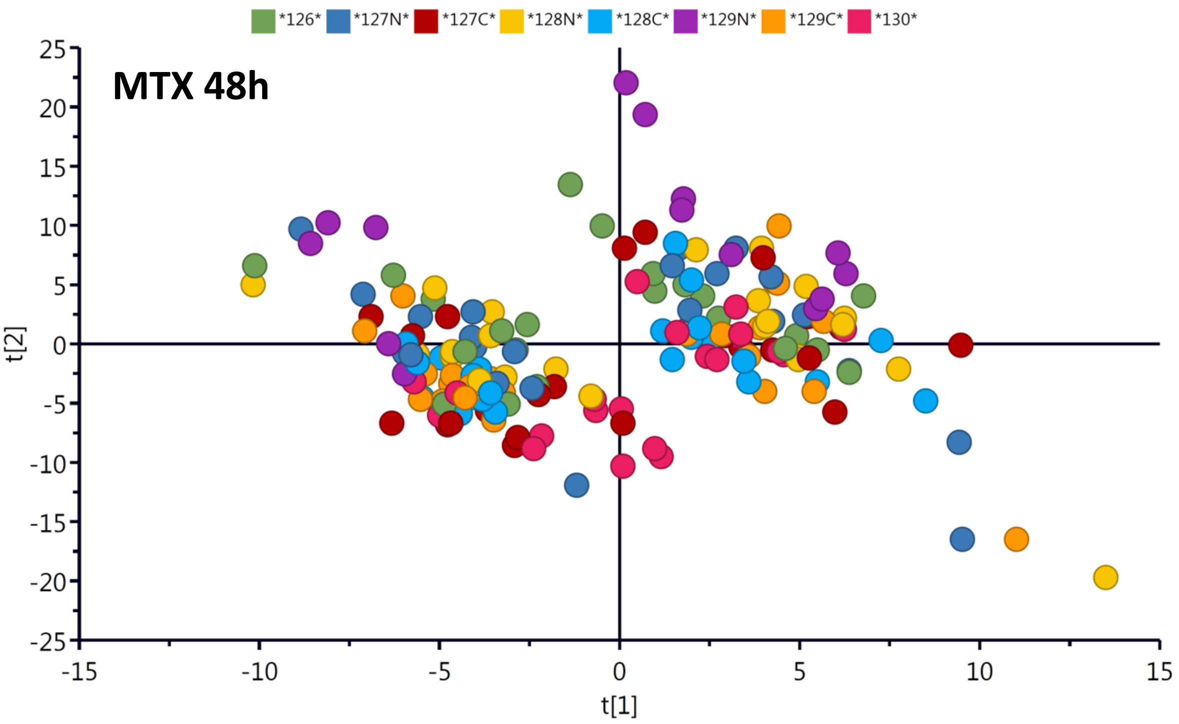

Figure S6. Clustering of the proteomes of attached treated single cells provides two groups of cells, G1 and G2.

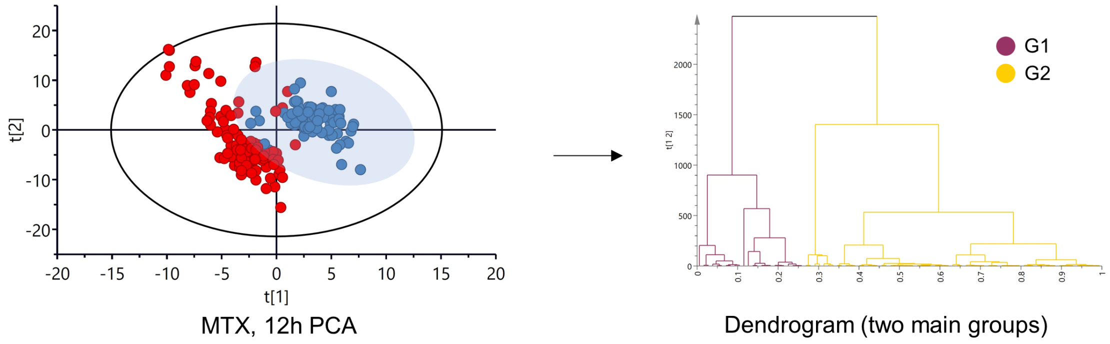

Figure S7. Same as in Figure S4, coloring the treated cells after the G1 and G2 subpopulations (depicted in red and blue colors, respectively; while untreated cells are shown as empty circles).

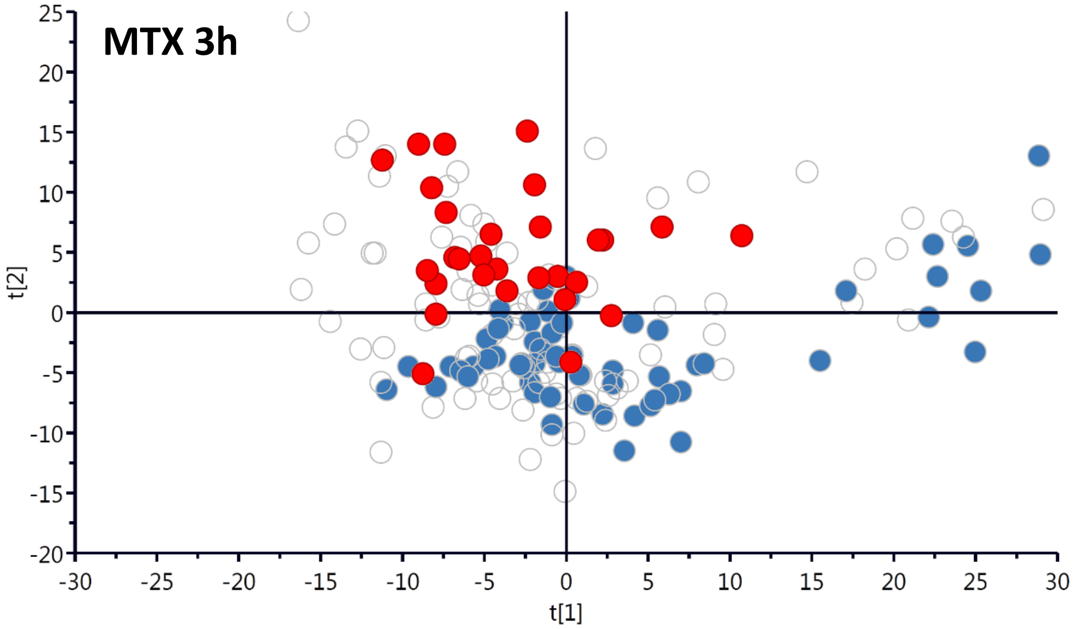

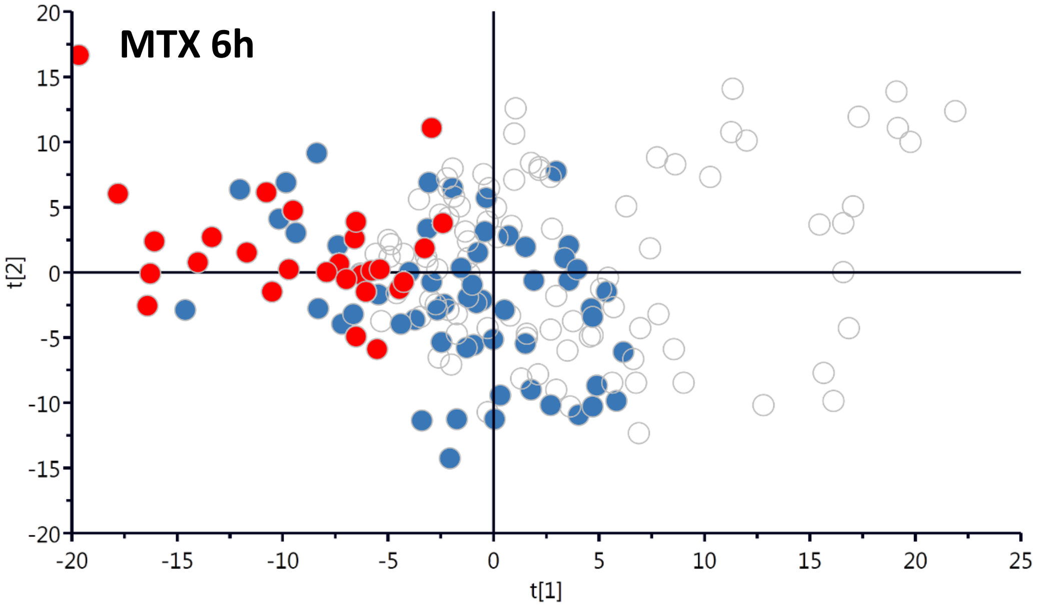

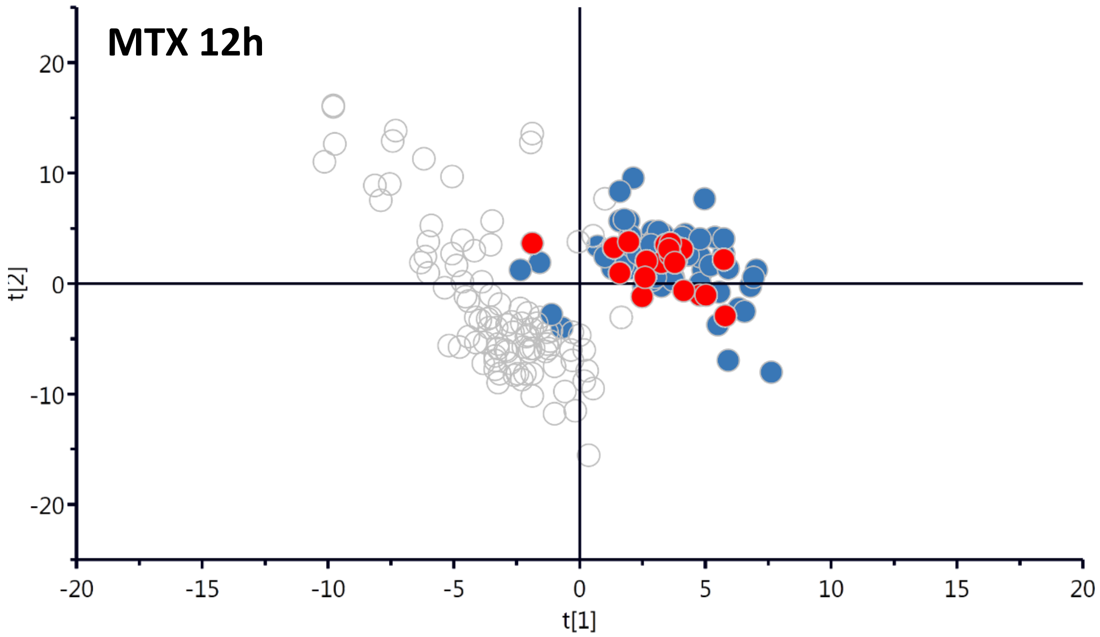
MTX 12h
20
10
0
-10
-20
t[2]
-20
-15
-10
-5
0
5
10
15
20
t[1]

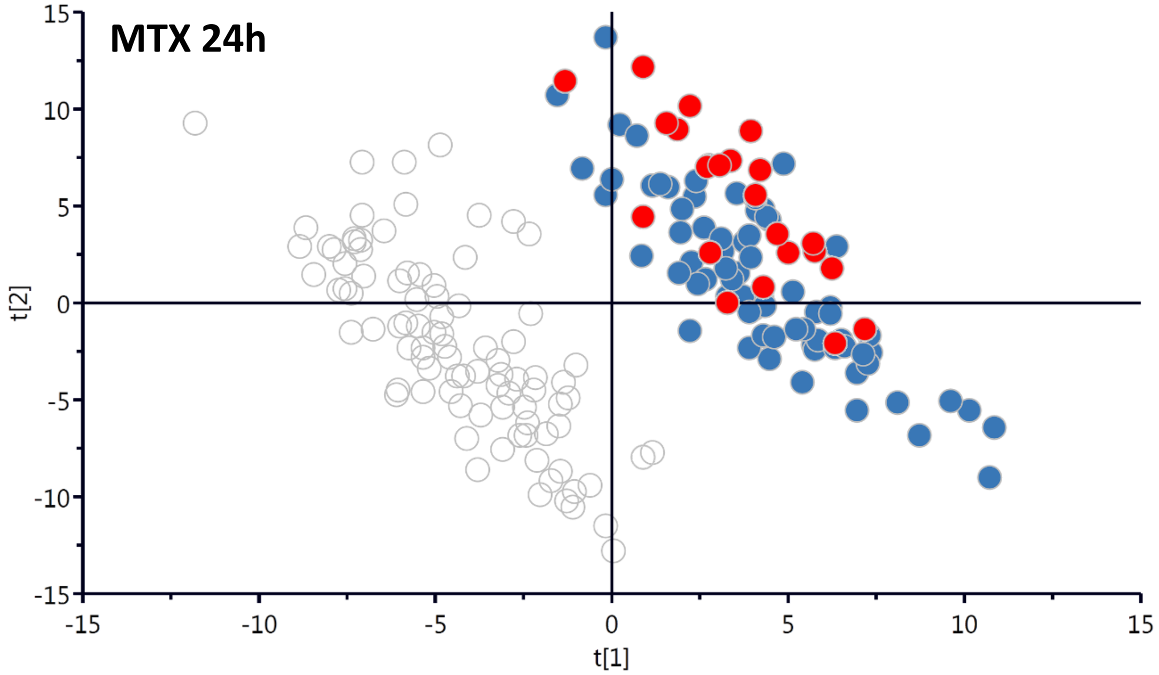
MTX 24h
15
10
5
0
-5
-10
-15
t[2]
-15
-10
-5
0
5
10
15
t[1]

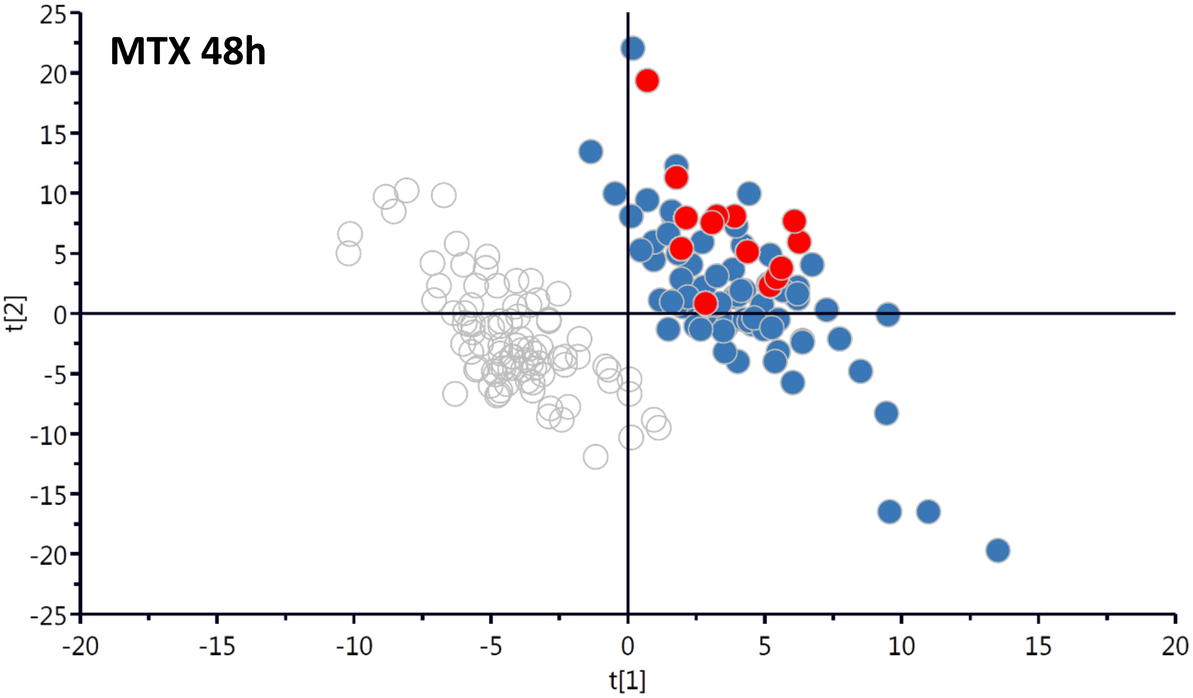
MTX 48h
t[2]
t[1]
25
20
15
10
5
0
-5
-10
-15
-20
-25
-20
-15
-10
-5
0
5
10
15
20

Figure S8. Volcano plots of data presented in *Figures 2* and *4*, with proteins re-colored according to their relative abundance in the sample (low to high proteins abundance = red-yellow-blue).

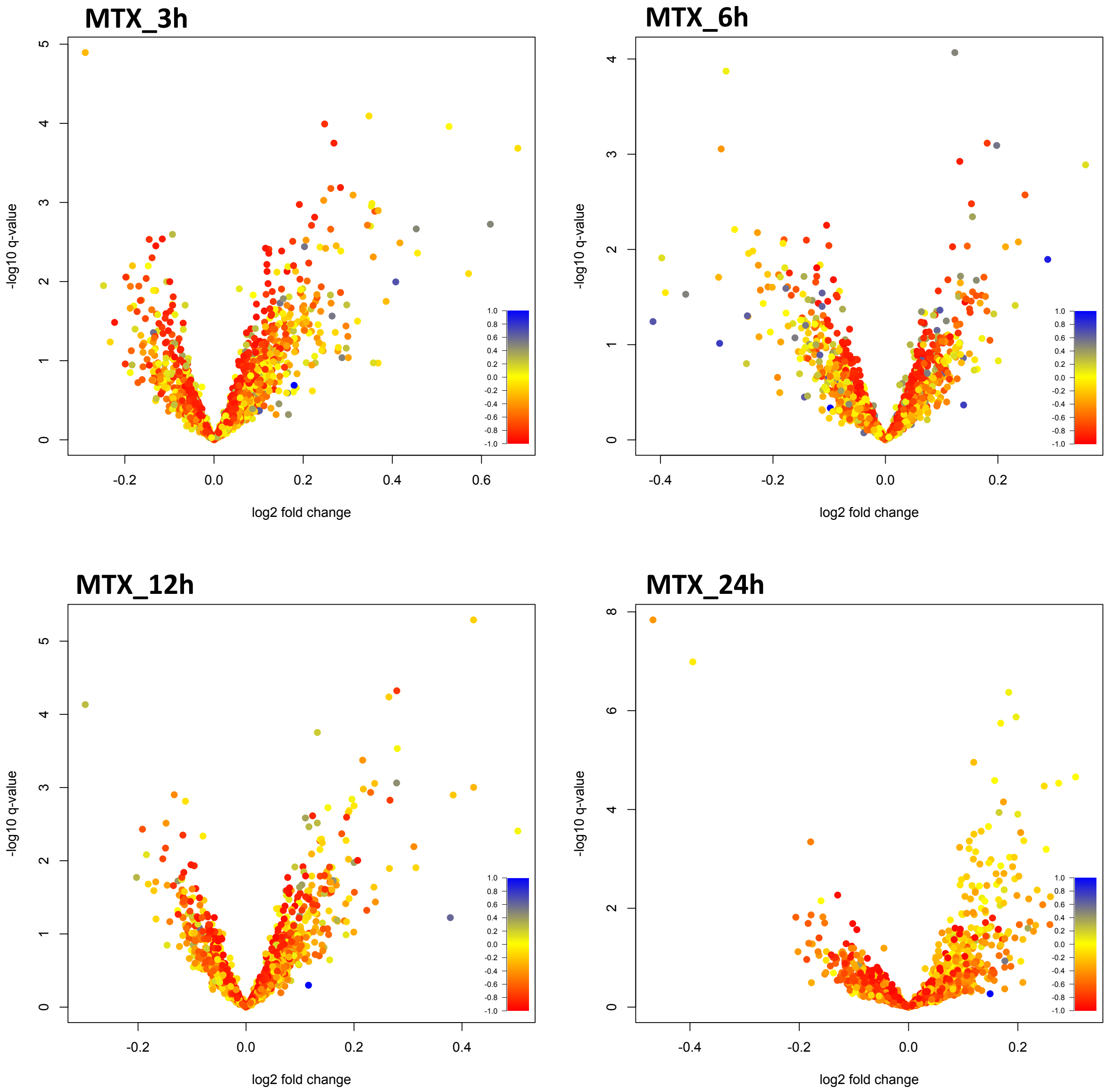

MTX_48h

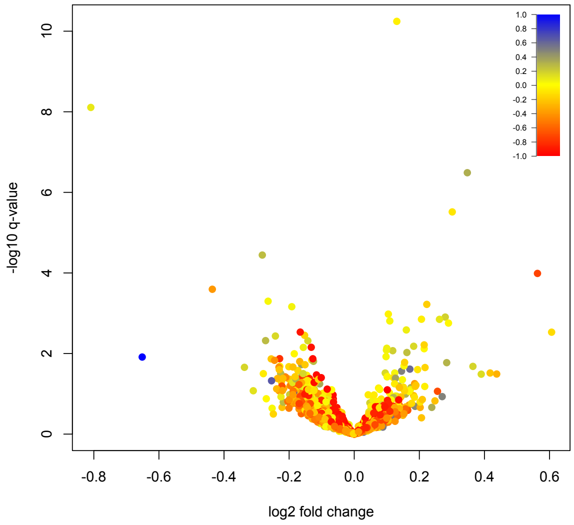

CPT_12h

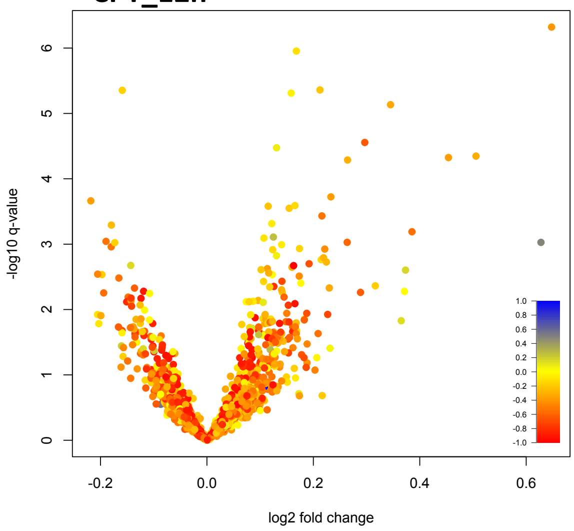

CPT_24h

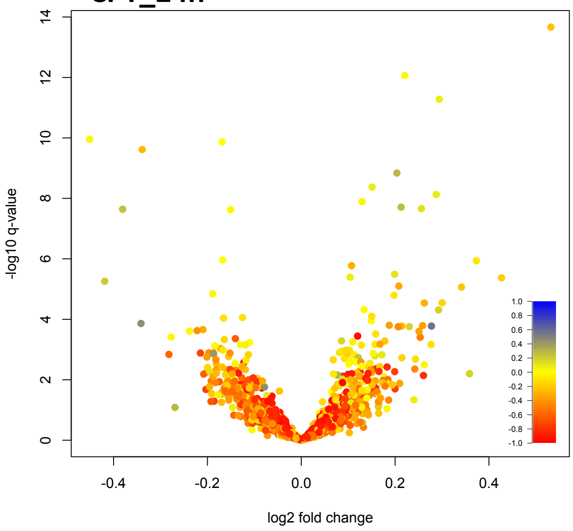

CPT_48h

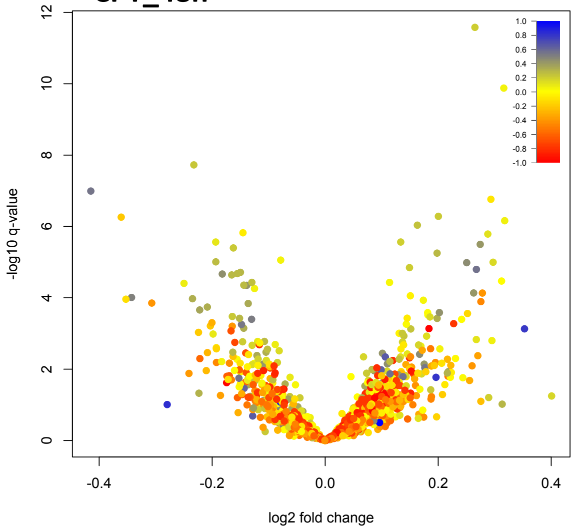

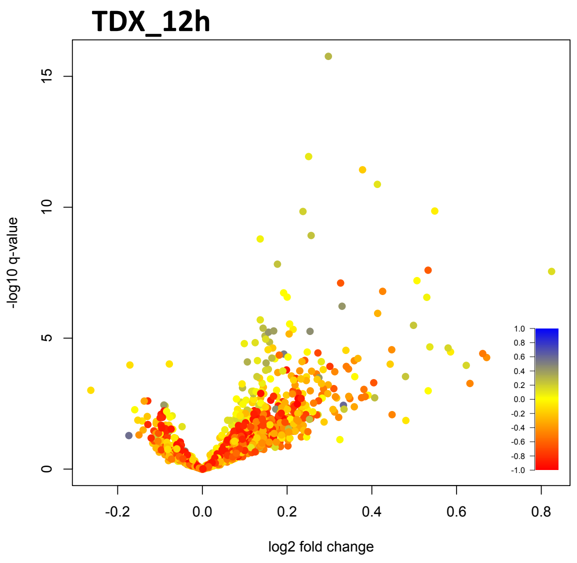
TDX_12h
-log10 q-value
log2 fold change

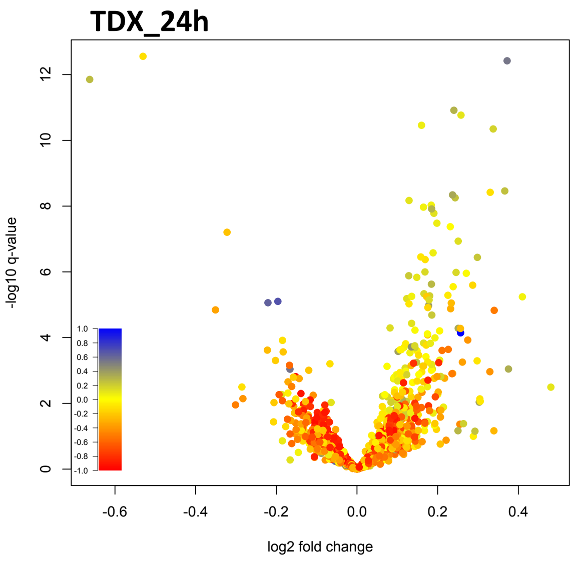
TDX_24h
-log10 q-value
log2 fold change

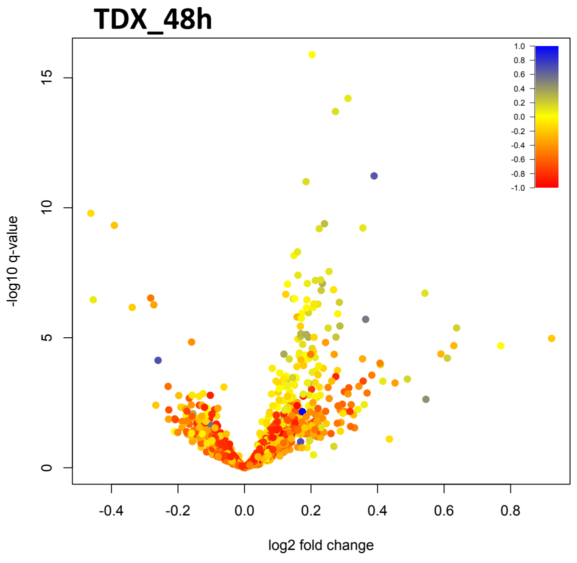
TDX_48h
-log10 q-value
log2 fold change

Figure S9. Proteins ranked after their abundances in data sets shown in *Figures 2* and *4*. The significantly regulated proteins are depicted in red color.

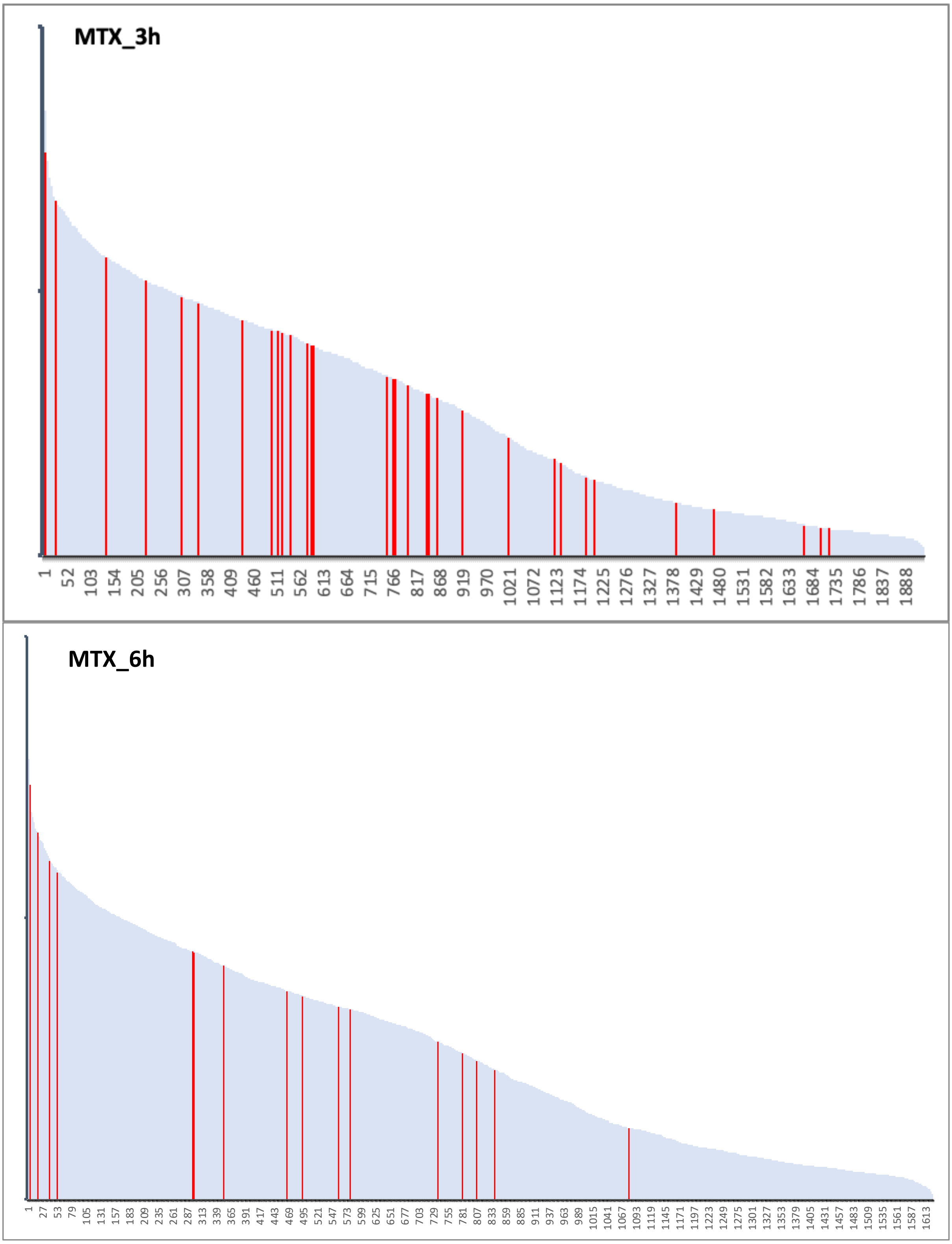

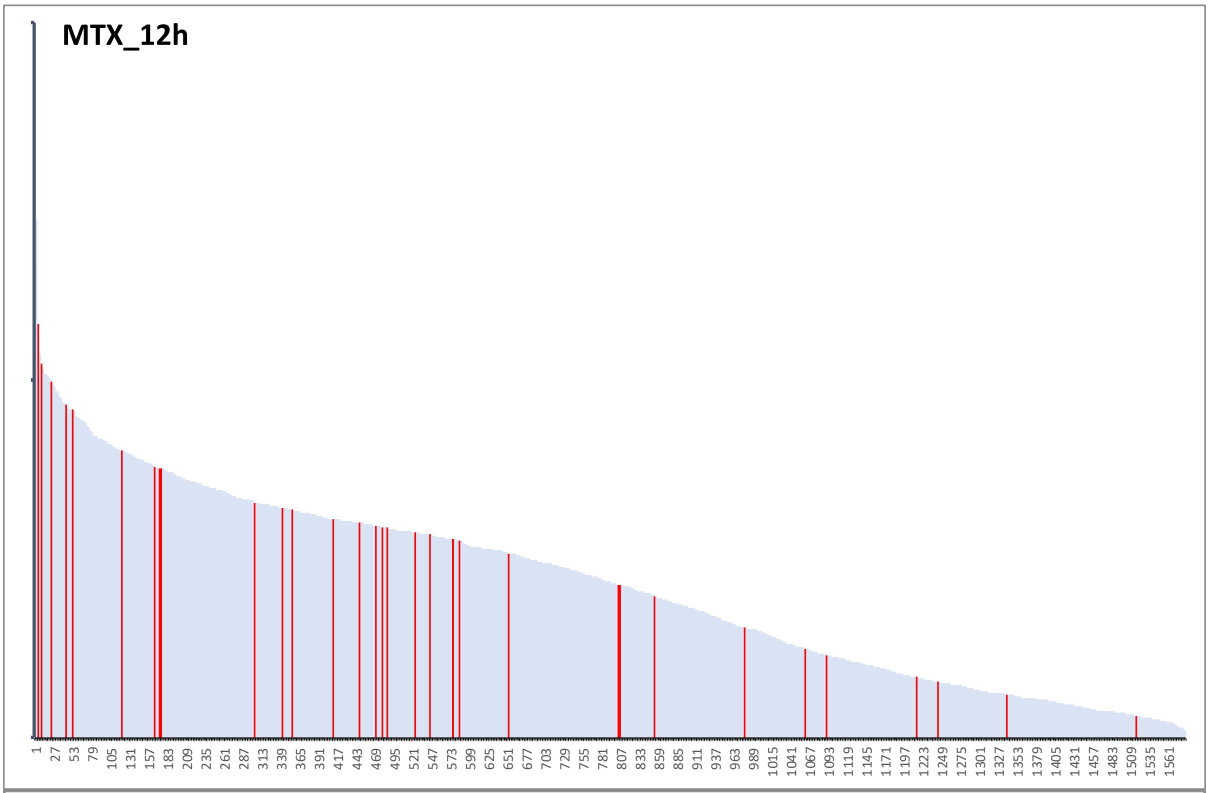
MTX_12h
1
27
53
79
105
131
157
183
209
235
261
287
313
339
365
391
417
443
469
495
521
547
573
599
625
651
677
703
729
755
781
807
833
859
885
911
937
963
989
1015
1041
1067
1093
1119
1145
1171
1197
1223
1249
1275
1301
1327
1353
1379
1405
1431
1457
1483
1509
1535
1561

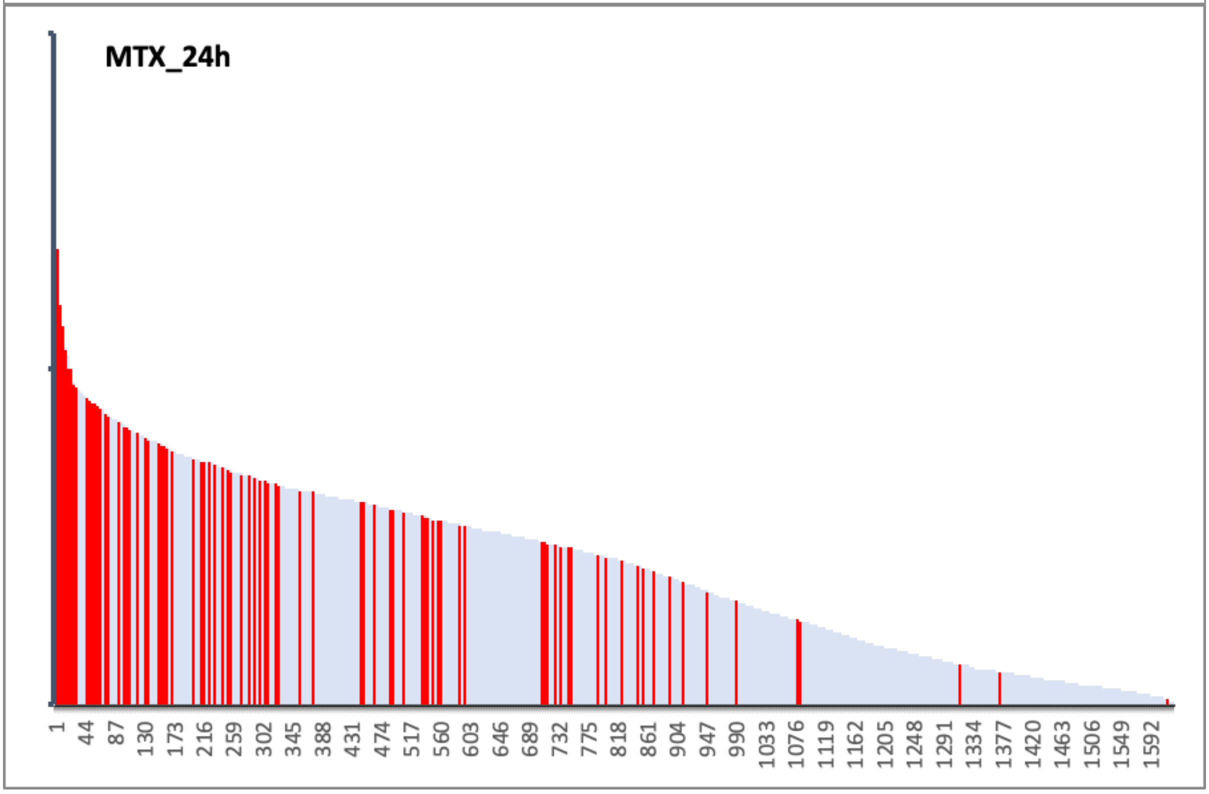
MTX_24h
1
44
87
130
173
216
259
302
345
388
431
474
517
560
603
646
689
732
775
818
861
904
947
990
1033
1076
1119
1162
1205
1248
1291
1334
1377
1420
1463
1506
1549
1592

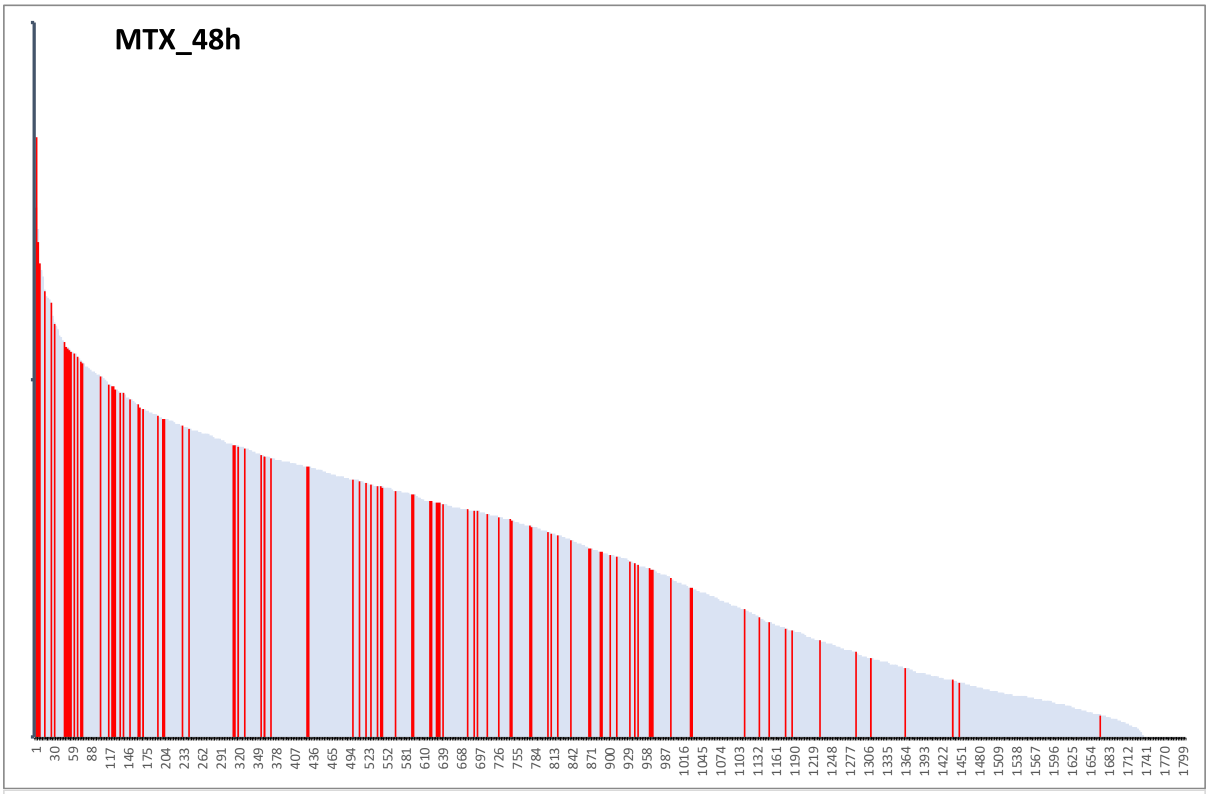
MTX_48h
1 30 59 88 117 146 175 204 233 262 291 320 349 378 407 436 465 494 523 552 581 610 639 668 697 726 755 784 813 842 871 900 929 958 987 1016 1045 1074 1103 1132 1161 1190 1219 1248 1277 1306 1335 1364 1393 1422 1451 1480 1509 1538 1567 1596 1625 1654 1683 1712 1741 1770 1799

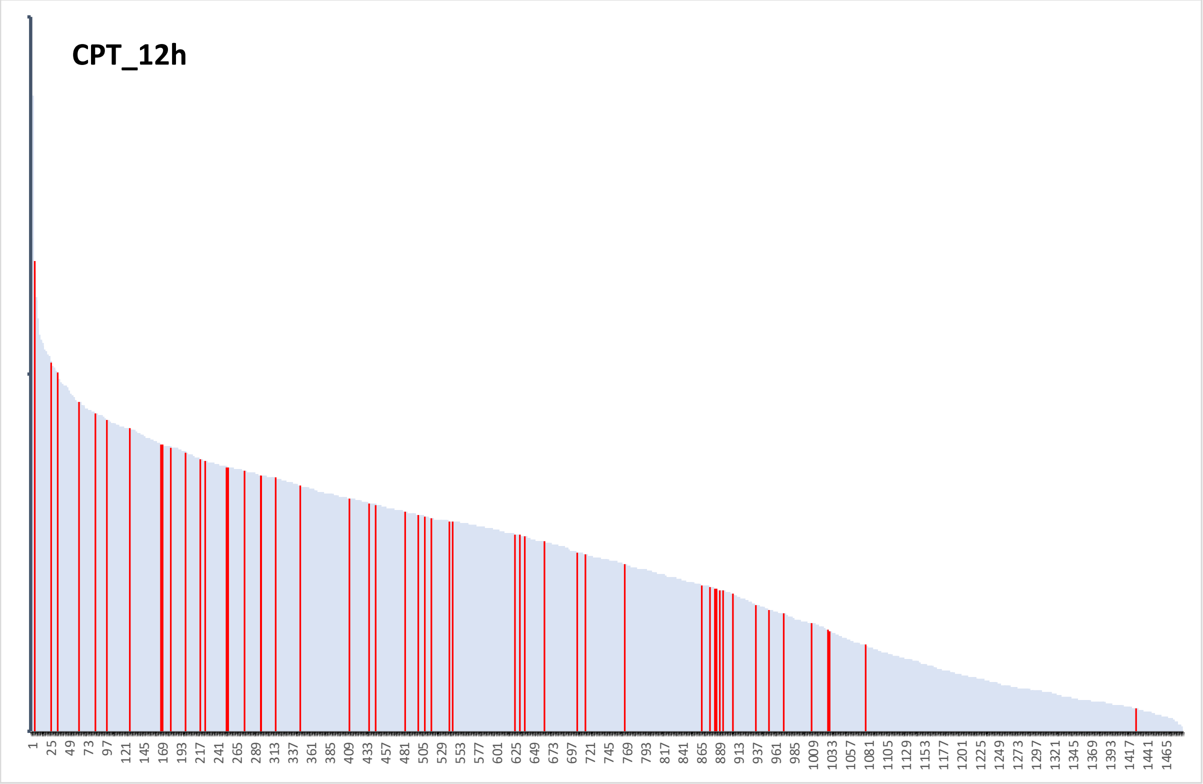
CPT_12h
1 25 49 73 97 121 145 169 193 217 241 265 289 313 337 361 385 409 433 457 481 505 529 553 577 601 625 649 673 697 721 745 769 793 817 841 865 889 913 937 961 985 1009 1033 1057 1081 1105 1129 1153 1177 1201 1225 1249 1273 1297 1321 1345 1369 1393 1417 1441 1465

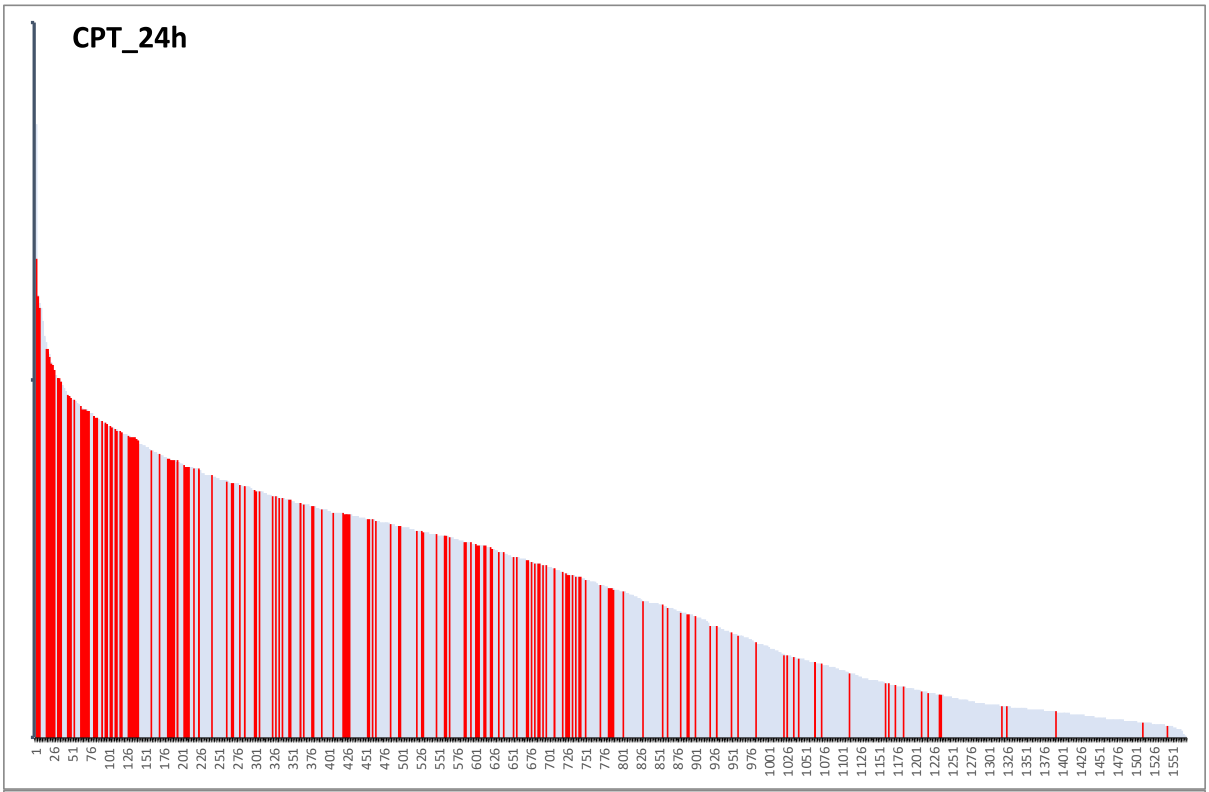
CPT_24h

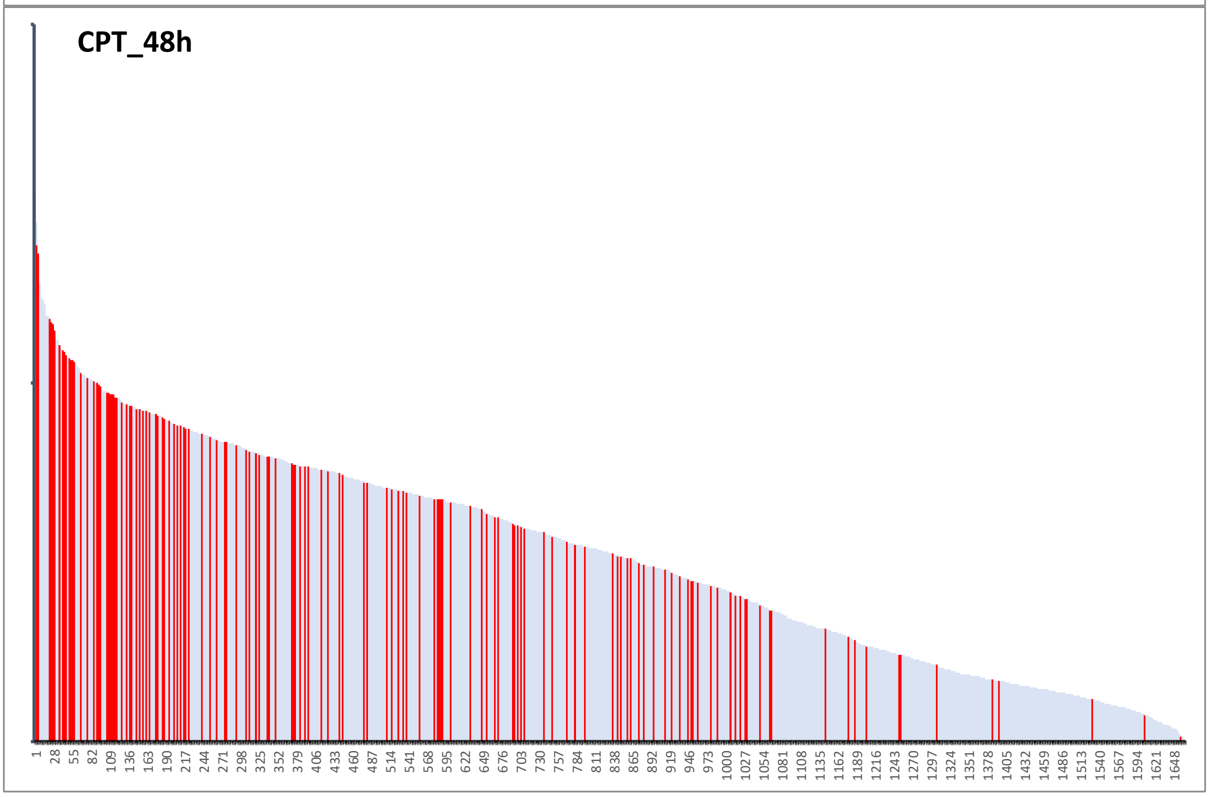
CPT_48h

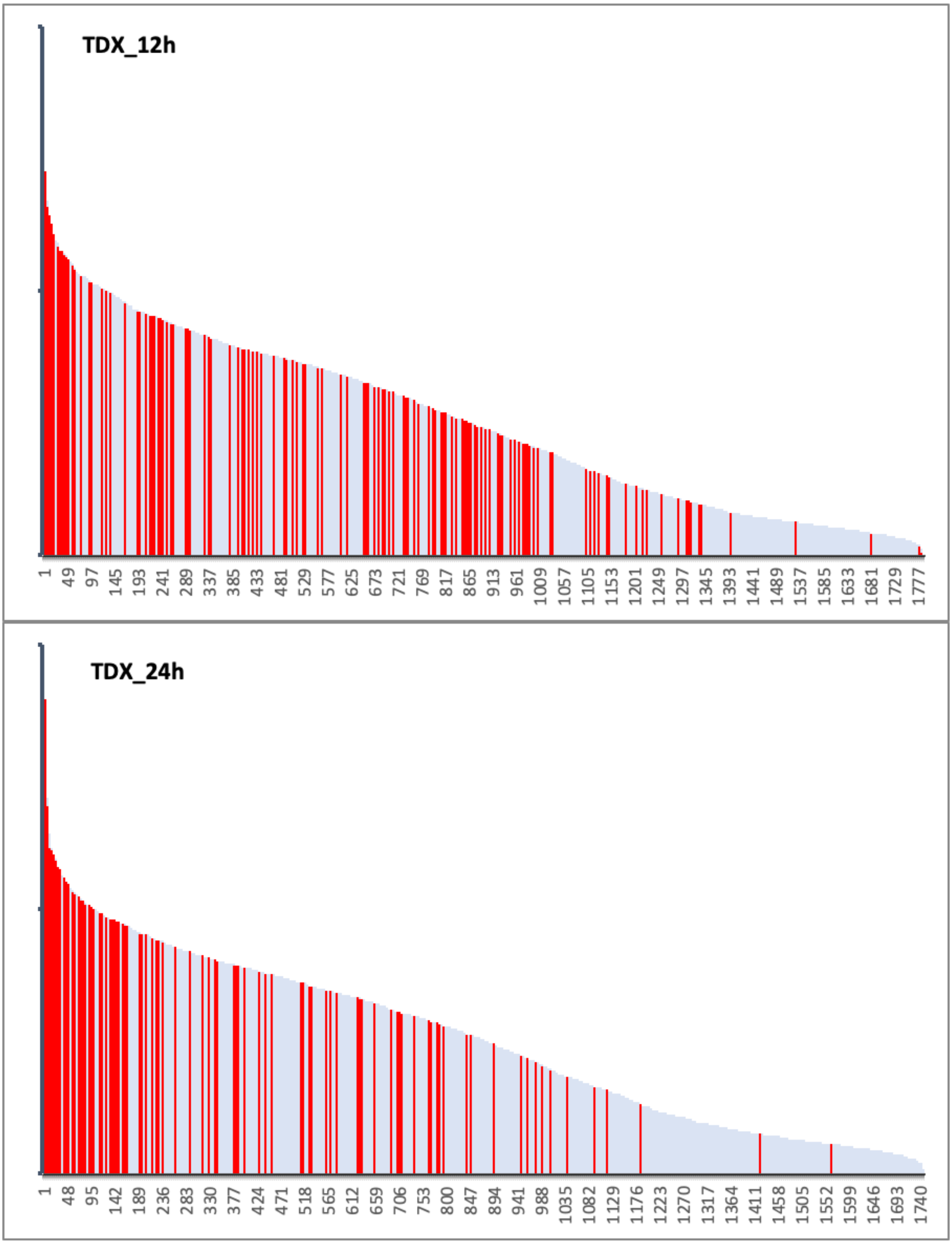
TDX_12h
1 49 97 145 193 241 289 337 385 433 481 529 577 625 673 721 769 817 865 913 961 1009 1057 1105 1153 1201 1249 1297 1345 1393 1441 1489 1537 1585 1633 1681 1729 1777
TDX_24h
1 48 95 142 189 236 283 330 377 424 471 518 565 612 659 706 753 800 847 894 941 988 1035 1082 1129 1176 1223 1270 1317 1364 1411 1458 1505 1552 1599 1646 1693 1740

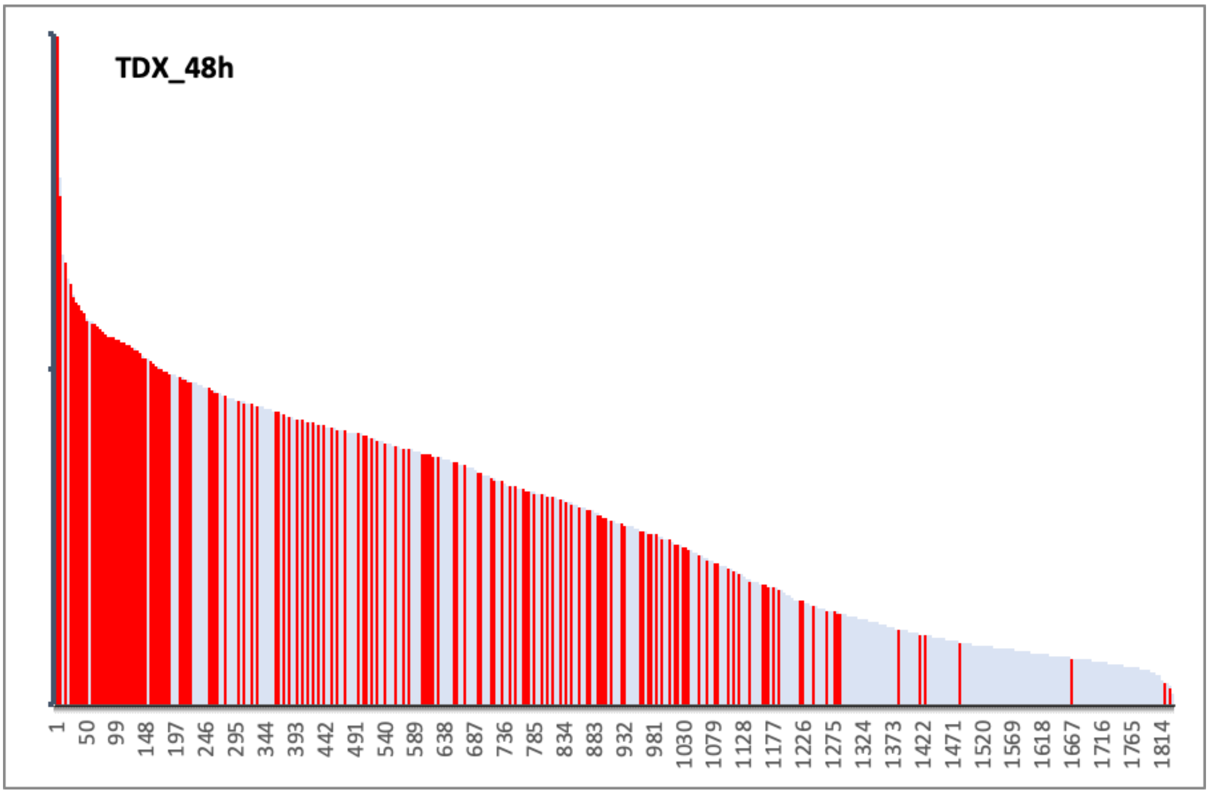

TDX_48h
1
50
99
148
197
246
295
344
393
442
491
540
589
638
687
736
785
834
883
932
981
1030
1079
1128
1177
1226
1275
1324
1373
1422
1471
1520
1569
1618
1667
1716
1765
1814

Figure S10. A) PCA plot of all single cell proteomics data acquired with MTX-treated and control cells. The cells were color-coded according to their treatment time and treatment status (see legend). B) The histogram of cells' first principal component (t[1]) for different treatment times.

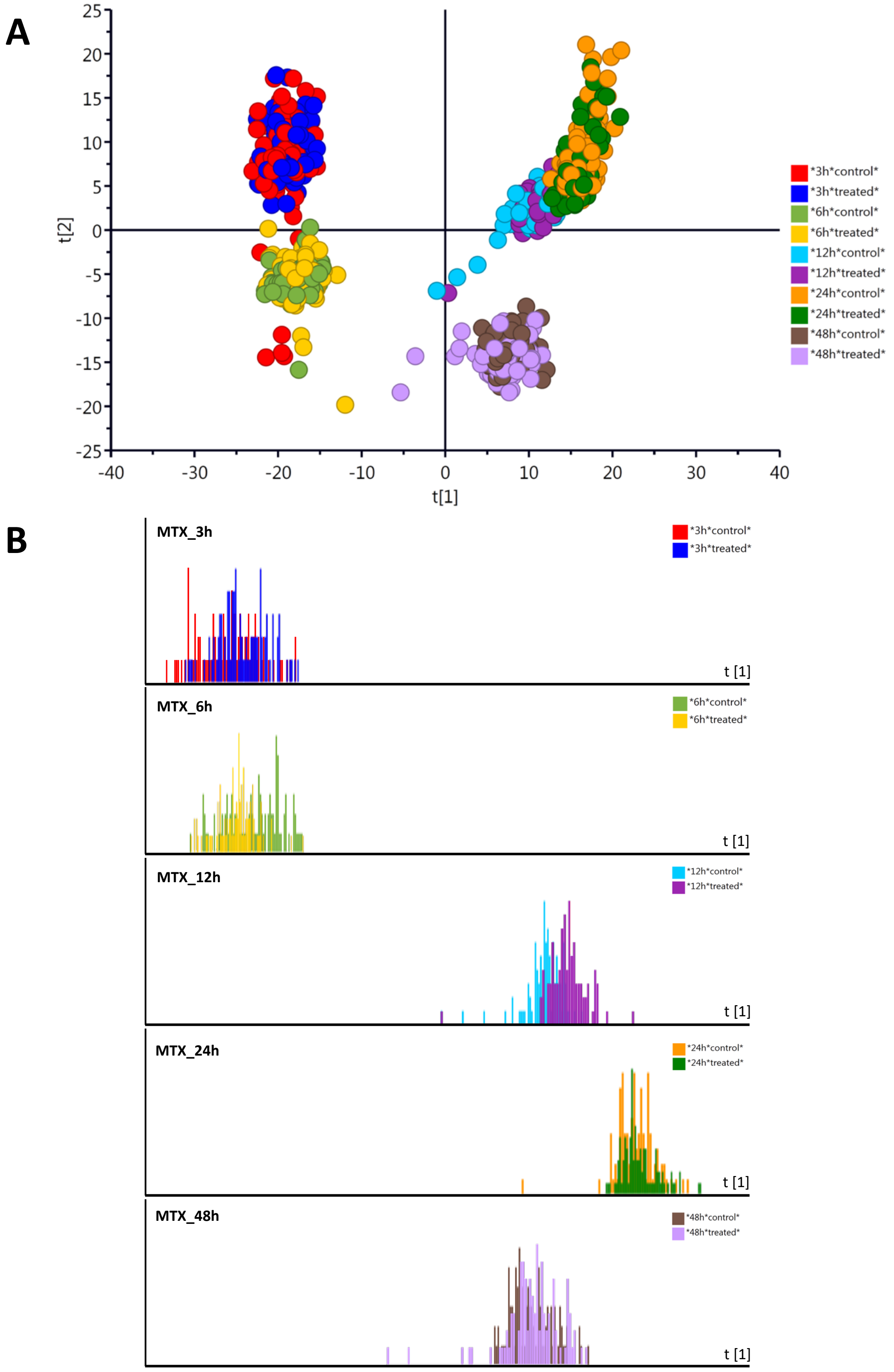

Figure S11. Pathway analysis of 179 proteins with significantly different abundances in G1 versus G2 subpopulations at 12 h past MTX treatment revealed that they preferentially belong to metabolic pathways and carbon metabolism (enriched in G2), as well as ribosome- and proteasome-related pathways (enriched in G1).

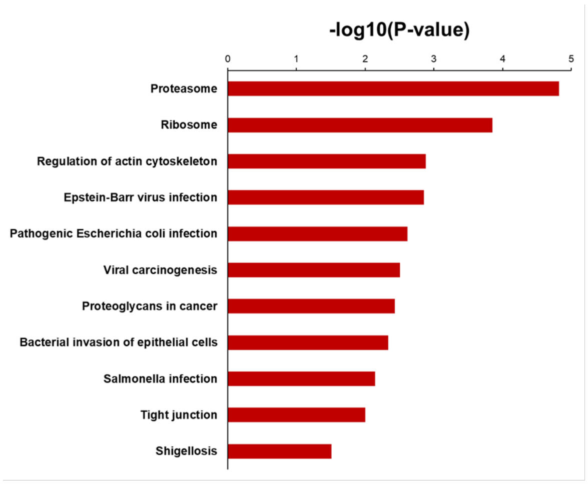

Pathways enriched in G1 (101 proteins)

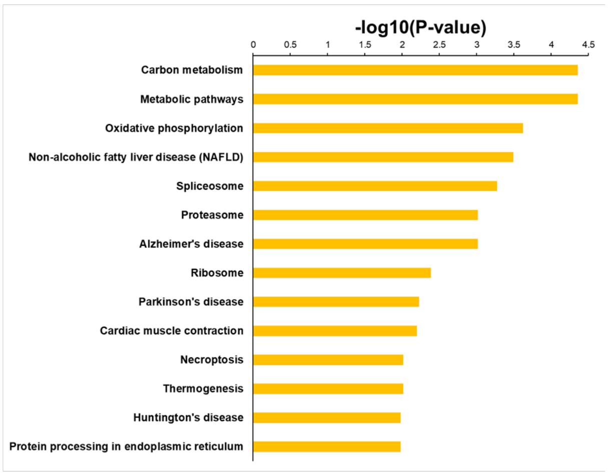

Pathways enriched in G2 (78 proteins)
